# Mechanical Stimulation on Mesenchymal Stem Cells and Surrounding Microenvironments in Bone Regeneration: Regulations and Applications

**DOI:** 10.3389/fcell.2022.808303

**Published:** 2022-01-21

**Authors:** Yuyang Sun, Ben Wan, Renxian Wang, Bowen Zhang, Peng Luo, Diaodiao Wang, Jing-Jun Nie, Dafu Chen, Xinbao Wu

**Affiliations:** ^1^ Laboratory of Bone Tissue Engineering, Beijing Laboratory of Biomedical Materials, Beijing Research Institute of Traumatology and Orthopaedics, Beijing Jishuitan Hospital, Beijing, China; ^2^ Department of Oral and Maxillofacial Surgery/Pathology, Amsterdam UMC and Academic Center for Dentistry Amsterdam (ACTA), Vrije Universiteit Amsterdam (VU), Amsterdam Movement Science (AMS), Amsterdam, Netherlands; ^3^ Department of Joint Surgery, Peking University Ninth School of Clinical Medicine, Beijing Shijitan Hospital, Capital Medical University, Beijing, China

**Keywords:** mechanical stimulations, mesenchymal stem cells, bone regeneration, mechanotransduction, immunomicroenvironment, angiogenesis

## Abstract

Treatment of bone defects remains a challenge in the clinic. Artificial bone grafts are the most promising alternative to autologous bone grafting. However, one of the limiting factors of artificial bone grafts is the limited means of regulating stem cell differentiation during bone regeneration. As a weight-bearing organ, bone is in a continuous mechanical environment. External mechanical force, a type of biophysical stimulation, plays an essential role in bone regeneration. It is generally accepted that osteocytes are mechanosensitive cells in bone. However, recent studies have shown that mesenchymal stem cells (MSCs) can also respond to mechanical signals. This article reviews the mechanotransduction mechanisms of MSCs, the regulation of mechanical stimulation on microenvironments surrounding MSCs by modulating the immune response, angiogenesis and osteogenesis, and the application of mechanical stimulation of MSCs in bone regeneration. The review provides a deep and extensive understanding of mechanical stimulation mechanisms, and prospects feasible designs of biomaterials for bone regeneration and the potential clinical applications of mechanical stimulation.

## 1 Introduction

Bone has extraordinary healing potential. However, approximately 5–10% of fractures cause fracture nonunion, partly because of large segmental bone defects (Holmes, 2017). Autologous transplantation of bone, though considered as a typical strategy for bone defect treatment, has shortages of limited autografts and donor-site morbidity, while the allogeneic bone graft is constrained by immune rejection (Hunziker, 2002). Therefore, tissue-engineered bone is a promising alternative to autologous bone grafting in the future. Although stem cell therapy is widely used in the bone regeneration field, the accurate regulation of stem cells remains a significant challenge. Traditional methods induce stem cells to the ontogenetic lineage by delivering biochemical signaling molecules such as growth factors. However, the difficulties in maintaining physiological concentration gradients and controlling the release of growth factors temporally and spatially have not yet been resolved. Therefore, regulating the differentiation of stem cells through physical means (such as mechanical stimulation) deserves further study.

Organs of the locomotor system undertake continuous mechanical loading, including compression on the bone, the stretch on muscles, and the fluid shear stress on blood vessels. Mechanical stimulation with different amplitudes, modalities, and durations plays an essential role in cell growth and differentiation, providing the possibility to regulate the lineage commitment of stem cells (Horner et al., 2019; McDermott et al., 2019; Ruehle et al., 2020). Mechanobiology is an emerging field specializing in the cellular response to mechanical cues, including the reception of mechanical signals and transduction of extracellular mechanical signals into intracellular biological signals (Fu et al., 2020). Cells can respond to pericellular mechanical stimulation from external mechanical stimulation and the properties of extracellular matrix (ECM). The process that cells convert exogenous mechanical signals into biochemical signals is called mechanical transduction (Dewey et al., 1981). Superficial mechanoreceptors of cells sense the mechanical cues, which are subsequently transmitted to the nucleus *via* the actin skeleton or chemical pathways. The nucleus responds to these signals by upregulating or downregulating the expression of genes related to mechanical stimulation (Kirby and Lammerding, 2018).

Mesenchymal stem cells (MSCs) are pluripotent cells that originate from intermediate mesoderm. MSCs have the potential to differentiate into lineages, including osteoblasts, adipocytes, chondrocytes, and myocytes. In the skeleton system, MSCs reside in bone marrow and periosteum. As one of the main functional cells in bone regeneration, MSCs enhance the bone healing process through cell-cell contact and secretion of growth factors such as BMP and VEGF (Charoenpanich et al., 2014; Schreivogel et al., 2019). Endochondral ossification is the bone regeneration mechanism involved in most fractures (Einhorn and Gerstenfeld, 2015). The bone defect first triggers an inflammatory process, which leads to the recruitment of mesenchymal stem cells (MSCs) to the bone defect by inflammatory factors. These MSCs then differentiate into cartilage that gradually ossifies with the growth of blood vessels into the cartilage model. Thus, MSCs play a crucial role in bone regeneration. MSCs regulate the immuno-microenvironment by interacting with macrophages and regulating blood vessel formation by secreting angiogenic growth factors. This process involves interacting cells, including MSCs, macrophages, and vascular endothelial cells, as well as extracellular matrix molecules and cytokines, all of which constitute the MSC niche that is of great significance in regulating bone regeneration (Moore and Lemischka, 2006; Kuhn and Tuan, 2010; Vafaei et al., 2017).

Previous studies have indicated that MSC differentiation was determined by the MSC niches (Chen et al., 2020). Moreover, recent studies have shown that MSC differentiation was also affected by mechanical stimulation (Ravichandran et al., 2017). A thorough understanding of the effect of mechanical stimulation on MSC niches in bone regeneration is of great value for establishing an *in vitro* model of bone regeneration and rehabilitation training of patients after fracture surgery. Therefore, this article reviews the intracellular mechanisms by which MSCs sense and respond to mechanical stimulation, the effect of mechanical stimulation on regulating MSC surrounding microenvironments by modulating the immune, angiogenic, and osteogenic microenvironments, and the applications of mechanical stimulation in bone regeneration.

## 2 Mechanism of Mesenchymal Stem Cell Sensing and Responding to Mechanical Stimulation

Mechanical stimulation plays an essential role in various physiological processes of bone. Wolff’s Law demonstrates that mechanical stimulation remolds the morphology of bone by the force line direction (Lanyon and Baggott, 1976; Woo et al., 1981). Bone mass increases in high stress regions and decreases in low stress regions. Wolff’s Law indicates that bone can sense and respond to the external mechanical loading and adapt to it by regulating bone metabolism. The lack of loading leads to disuse osteoporosis in the clinic, which explains why bedridden patients suffer from bone loss (Qi et al., 2012). Several types of bone cells can sense mechanical stimulation, including bone marrow MSCs and osteocytes. These cells function in different physiological processes and respond to external mechanical stimuli.

### 2.1 Mechanism of Mesenchymal Stem Cells Sensing Mechanical Stimulation

#### 2.1.1 Physiological Basis

It is widely accepted that osteocytes are mechanosensitive cells that respond to mechanical stimulation (Yan et al., 2020). However, recent studies proves that external mechanical stimulation regulates bone marrow mesenchymal stem cells (BMSCs) toward osteogenic lineage which is independent of osteocytes regulation (Schreivogel et al., 2019).

The lacunar-canalicular system (LCS) is filled with interstitial fluid (Timmins and Wall, 1977). Intramedullary pressurization alteration and deformation of bone matrix generate interstitial fluid flow (Kwon et al., 2010; Price et al., 2011; Ciani et al., 2014). Therefore, mechanical loading leads to variation in intramedullary pressurization, which results in shear stress generation. Shear stress applies to osteocytes in LCS and MSCs in the bone marrow. Fluid shear stress is the general form of the force applied to MSCs in the bone marrow under physiological conditions (Gurkan and Akkus, 2008). The form of the force applied to MSCs in the periosteum is mainly caused by micro-deformation of bone generated by external mechanical stimuli such as stretching and compression. MSCs respond to the stimulation indirectly by sensing the micro-deformation of the extracellular matrix. Therefore, when investigating the mechanism of the mechanical loading effect on MSC differentiation, the function of both the direct and indirect force ought to be considered.

#### 2.1.2 Mechanosensors

**FIGURE 1:**
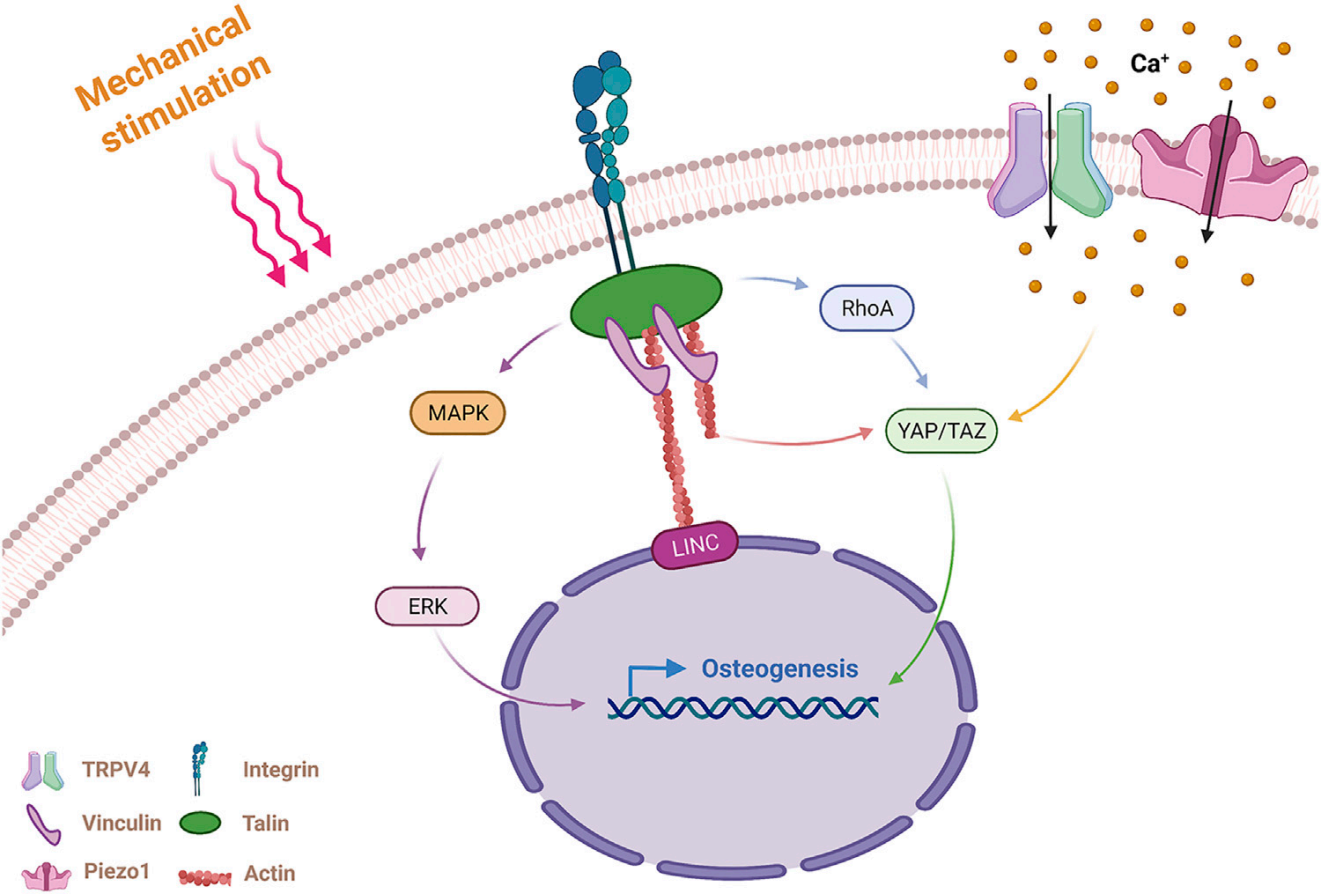
Mechanism of MSCs sensing and responding to mechanical stimulation. MSCs sense external mechanical stimulation via integrins and mechanosensitive ion channels and transmit the mechanical signals *via* actin stress fibers and molecular pathways. Integrins activate RhoA, MAPK pathways, and actin fibers by FAs (including vinculin and talin) in response to mechanical stimulation. MAPK promotes osteogenesis through nuclear localization of ERK. The RhoA pathway and actin fibers promote osteogenesis through nuclear localization of YAP/TAZ. The mechanosensitive ion channels TRPV4 and Piezo1 generate an intracellular Ca^2+^ influx after sensing mechanical stimulation, and Piezo1 promote osteogenic differentiation through nuclear localization of YAP/TAZ. MSCs, mesenchymal stem cells; RhoA, Ras homolog gene family, member A; MAPK, Mitogen-activated protein kinases; FA, focal adhesion; ERK, extracellular signal-regulated kinase; YAP/TAZ, Yes-associated protein/transcriptional coactivator with PDZ-binding motif; TRPV4, transient receptor potential vanilloid 4.

The cellular response to external mechanical stimuli involves two processes: mechanosensing and mechanotransduction (Argentati et al., 2019). Mechanosensing is the process by which cells sense physical signals from the extracellular environment by mechanoreceptors. Cells then transduce the physical signals into biochemical signals. This process results in differentiation of the cells to specific lineages and is known as mechanotransduction (Delaine-Smith and Reilly, 2011). Several typical mechanoreceptors present on the membrane are introduced here, including integrins, mechanosensitive ion channels and primary cilia (Figure 1).

##### 2.1.2.1 Integrin

Integrins, widely recognized mechanical sensors, are transmembrane proteins that can take up physical signals from the ECM (Kechagia et al., 2019). Integrins mediate the adhesion and transmit the mechanical signal between cells and the ECM. One end of the integrin connects to the ligands (proteins of the extracellular matrix), and the other end connects to the intracellular actin fiber via adaptor proteins. Actin stress fiber senses mechanical signals originating from the ECM by the degree of its contraction. The link containing ECM, integrins, adaptor proteins and actin transmitting mechanical cues is known as clutches. External mechanical signals exert mechanical force on actin that tunes the integrins’ alignment and reorders the actin cytoskeleton (Kechagia et al., 2019). The interactions between the ECM and the cytoskeleton alter the cells lineage and lead to remodeling of the ECM (Loebel et al., 2019).

Cells perceive external stimulation from the ECM and transmit mechanical signals to the nucleus to regulate gene expression. The adapter proteins that connect integrins and actin fibers include focal adhesion (FA) molecules, which are mainly composed of vinculin, paxillin, talin and focal adhesion kinase (FAK). The Rho and MAPK signaling pathways activated by FA lead to nuclear localization of the transcription factors Yes-associated protein/transcriptional coactivator with PDZ-binding motif (YAP/TAZ) and ERK, respectively (Nardone et al., 2017). In addition to the means of transmitting mechanical signals by chemical signals, the nuclear envelope and Linker of Nucleoskeleton and Cytoskeleton (LINC) complex also play essential roles in mechanotransduction (Bouzid et al., 2019).

##### 2.1.2.2 Mechanosensitive Ion Channels

Studies have shown that mechanical stimulation partly impacted the concentration of intracellular calcium ions. Intracellular calcium ions of pre-osteoblasts rapidly increase under stimulation by fluid shear stress, possibly as a result of the activation of mechanically sensitive calcium channels on cells (Chen et al., 2000). Osteoblasts contain several calcium channels, including transient receptor potential vanilloid 4 (TRPV4), multimeric L-type and T-type voltage-gated calcium channels (VSCC), and the recently discovered mechanically sensitive ion channel Piezo1. Both TRPV4 and Piezo1 are mechanically sensitive ion channels.

Ten years ago, Bertrand reported that the Piezo1 channel was a mechanically activating cation channel (Coste et al., 2010). Later, it was found that Piezo1 sensed and transduced mechanical stimuli in various cells, including endothelial cells, neural stem cells and chondrocytes (Lee et al., 2014; Li et al., 2014; Pathak et al., 2014). Piezo1 also plays an important role in the response of skeleton cells to mechanical stimulation, and governs bone homeostasis by reacting to mechanical signals. Sugimoto et al. (2017) proved that hydrostatic pressure (HP) promoted bone formation and osteogenic differentiation of MSCs through the mechanically sensitive ion channel Piezo1, which was related to the expression of bone morphogenetic protein 2 (BMP-2). Li X. et al. (2019) discovered that bone cells could also sense and respond to changes in fluid shear stress via Piezo1. After fluid shear stress is applied to bone cells, the mechanically sensitive ion channel Piezo1 partially activates YAP1 and TAZ to increase the expression of Wnt1 and regulate bone formation. In addition to investigating the mechanism of Piezo1 activation by various types of mechanical stimuli, Wang et al. recently investigated the function of Piezo1 in regulating the bone remodeling process. In this study, conditional knockout of the Piezo1 gene was found to reduce the cortical thickness and the trabecular bone volume in mice. Further studies have explained the role of Piezo1 in osteoblasts during bone remodeling. The results indicated that Piezo1 regulated osteoclast differentiation by regulating the expression of YAP type II and type IV collagen (Wang et al., 2020). The results showed that Piezo1 played an important role in maintaining bone homeostasis by regulating the crosstalk between osteoblasts and osteoclasts under mechanical stimulation conditions.

TRPV4, another mechanically sensitive calcium ion channel in MSCs, primarily localizes in the high strain regions (especially the primary cilia). TRPV4’s principal function is to promote early bone formation under the stimulation of oscillatory fluid shear stress (Hu et al., 2017; Corrigan et al., 2018). Some studies have compared the roles of the two mechanically sensitive ion channels, TRPV4 and Piezo1, in sensing mechanical stimulation. Yoneda et al. found that when osteoblasts were stimulated by short term shear stress (5 s), the ion channel TRPV4 rather than Piezo1 mediated the sensing process to the mechanical stimulus (Yoneda et al., 2019). Another study of TRPV4 and Piezo1 channels in chondrocytes showed that TRPV4 channels mediated strain at the physiologic level, and Piezo2 mediated strain at the injurious level (Du G. et al., 2020). These results indicated that the magnitude and duration of shear stress required to activate the Piezo1 and TRPV4 channels of the osteoblast lineage are likely different. A recent study also showed that the activation of TRPV4 was regulated by the activation of Piezo1 in vascular endothelial cells (Swain and Liddle, 2021). However, a comprehensive comparison of the relationships between the mechanically sensitive ion channels TRPV4 and Piezo1 in osteoblast lineage has not yet been conducted.

##### 2.1.2.3 Primary Cilium

In addition to the above two mechanoreceptors, primary cilium plays an essential role in sensing and responding to fluid shear stress in MSCs. Primary cilium was first identified and observed in osteocytes more than 40 years ago (Federman and Nichols, 1974). A laboratory in Sweden stimulated human MSCs (hMSCs) with oscillatory fluid flow (OFF) *in vitro* to simulate the fluid shear stress in the physiological environment. The results showed that OFF promoted the proliferation of hMSCs, increased the expression of osteogenic genes, and demonstrated that primary cilia mediated the response of hMSCs to fluid shear stress stimulation (Hoey et al., 2012). This laboratory then found that the mechanically reactive G protein-coupled receptor (GPCR) GRP161, located on the primary cilium, activated adenylate cyclase 6 (AC6) to respond to stimulation generated by fluid shear stress. AC6 then activates the cAMP signal, which increases the expression of PTCH1 and GLI1 in the hedgehog pathway via upregulating the expression of osteogenic genes (Johnson et al., 2021). Some ion channels, including TRPV4, are also widely localized in primary cilia, mediating fluid shear stress-induced calcium signaling and osteogenic process of MSCs (Hu et al., 2017; Corrigan et al., 2018).

### 2.2 Molecular Mechanism of Mesenchymal Stem Cells Responding to Different Mechanical Stimulations

#### 2.2.1 Stretching

Previous studies have shown that mechanical stretching could promote the osteogenic differentiation of mesenchymal stem cells of multiple origins through several molecular pathways, such as BMSCs and adipose-derived stem cells (Wang et al., 2017; Fang et al., 2019). Tensile strain stimulation promotes MSC osteogenesis differentiation and inhibits differentiation toward adipogenesis mainly through the Smad signaling pathway (Li R. et al., 2015; Grier et al., 2017). The Hedgehog (Hh) signaling pathway plays an essential role in cyclic mechanical stretch (CMS). Wang et al. found that DNA methyltransferase 3b (Dnmt3b) inhibited the expression of Hedgehog signaling by binding to the Shh gene promoter to downregulate the sensitivity of MSCs to stretch stimulation (Wang et al., 2017). Jiali Tan et al. found that the osteogenic effect of mechanical stretch on MSCs was correlated with donor age. The osteogenic effect of MSCs responding to the mechanical stretch in young rats was higher than that in adult rats. Additionally, stretch also resulted in more production of ROS inhibited osteogenesis, in MSCs of adult rats than in young rats (Tan et al., 2015). However, Chen et al. suggested that appropriate levels of mechanical stretching not only promoted osteogenesis of BMSCs but also reduced ROS levels in BMSCs and induced antioxidant responses by activating the AMPK-SIRT1 pathway (Chen et al., 2018).

Stretch stimulation can also regulate the lineage differentiation of MSCs by modulating the expression of miRNA that regulating pathway molecules. Liu et al. identified differentially expressed miRNAs after stretch stimulation and found that miR-503-5p was downregulated. Therefore, it was concluded that miR-503-5p was a mechanosensitive miRNA, and miR-503-5p downregulation could promote stretch stimulation-induced osteogenic differentiation of BMSCs (Liu et al., 2017). Li J.’s (2015) work found that miR-154-5p negatively regulated the Wnt/PCP (Rhoa-Rock) pathway to induce osteogenesis of ADSCs (Li J. et al., 2015).

#### 2.2.2 Compression

Dynamic compression can promote the differentiation and mineralization of MSCs toward osteogenesis both *in vitro* and *in vivo*, which partially replaces the role of osteogenic induction medium (Duty et al., 2007; Baas et al., 2010; Sittichokechaiwut et al., 2010; Ravichandran et al., 2017). It has been shown that dynamic compression did not directly regulate the expression of transcription factors such as RUNX2, but rather promoted MSC osteogenic differentiation in an autocrine manner by increasing BMP expression (Schreivogel et al., 2019).

Previous studies have also indicated that compression could promote both osteogenesis and chondrogenesis of MSCs (Cao et al., 2019). However, the mechanism underlying the effect of compression stimulus on MSCs and the means of controlling the differentiation of MSCs has not yet been fully explored. Possible factors include the magnitude of compression, the induction mode, and pathway activation. A previous study by Efstathios suggested that the differentiation of MSCs was related to the magnitude of compression. The study found that hMSCs differentiated toward osteogenesis under 10% dynamic compression but toward chondrogenesis under 15% (Michalopoulos et al., 2012). Moreover, Christopher et al. found that osteogenic differentiation decreased with an increase in the compression magnitude in osteogenic induction medium (Horner et al., 2018). However, another study suggested that the compression-induced MSC differentiation toward chondrogenic or osteogenic lineages depended on the activation of the ERK1/2 pathway (Pelaez et al., 2012). Dynamic compression induces chondrogenic differentiation of MSCs under normal conditions and osteogenesis differentiation when the ERK1/2 pathway is inhibited.

#### 2.2.3 Fluid Shear Stress

MSCS residing in the periosteum and bone marrow are exposed to fluid shear stress generated by mechanical stimulation-induced deformation. Therefore, the osteogenic differentiation of MSCs induced by mechanical stimulation is also related to the fluid shear stress caused by cyclical hydrostatic pressure (CHP) *in vivo*. The ability of shear stress to promote osteogenesis of MSCs has been widely recognized, and shear stress can promote MSC osteogenesis in the absence of a chemical induction medium (Yourek et al., 2010; Yue et al., 2019). MSCs mediate fluid shear stress through primary cilia and mechanosensitive ion channels such as TRPV4 and Piezo1 (Hu et al., 2017; Johnson et al., 2018; Li X. et al., 2019). Although fluid shear stress is recognized as one of the biophysical means to promote osteogenesis, the application of shear force in bone tissue engineering requires further exploration. As Zhang et al. (2012) found in their study, MSCs from different patients showed inconsistent responses to shear stress stimulation, which may be due to the high heterogeneity of the samples. Therefore, future exploration should target at more specific populations, such as the response of osteoporotic populations MSCs to shear force.

#### 2.2.4 Vibration

Although vibration is not a sort of mechanical stimulation in physiological condition, a great number of studies have been conducted on the vibration in osteogenesis (Chen et al., 2015; Pongkitwitoon et al., 2016). As is convenient to be applied on tissue, vibration has been used in osteoporosis treatment (Jepsen et al., 2019). Vibration stimulates skeleton with the motion of the body. Vibrations of the appropriate magnitude and frequency can trigger anabolic responses in the bones (Minematsu et al., 2019). Low magnitude vibration (LMV) is widely accepted by doctors and patients in clinic as a measure of exercise therapy based on the vibration (Wysocki et al., 2011). Thus, it is necessary to explore the mechanism of vibration in bone regeneration.

Vibration regulates and coordinates MSC bone resorption and formation via multiple signaling pathways. Previous studies have shown that vibration regulated the Wnt signaling pathway to promote MSC osteogenesis (Gao et al., 2017). Chen et al. (2016) demonstrated that vibration increased the adhesion and osteogenesis of MSCs on HA-coated surfaces by activating the Wnt/β-catenin signaling pathway. They supposed that the vibration may provide a means to promote the osseointegration of bone implants. Vibration enhances β-catenin function through inhibiting the β-catenin destruction complex element GSK3β (glycogen synthase kinase 3β), which promotes the Linker of Cytoskeleton and Nucleoskeleton (LINC) function (Uzer et al., 2018). Another study found that the expression of miR-335-5p was upregulated *via* vibration. miR-335-5p induces osteogenic differentiation by suppressing the expression of Dickkopf-related protein 1, a Wnt signaling inhibitor (Zhao et al., 2019). In addition to the Wnt pathway, vibration can also regulate the bone formation process by up-regulating the expression of estrogen receptor α (Li H. et al., 2019). Estrogen receptor α is known to be a mediator in bone remodeling and is significant in estrogen-deprived osteoporotic (Jessop et al., 2004). ERK1/2 pathway and p38 MAPK signaling have also been shown to play an essential role in vibration-induced osteogenesis of MSCs (Zhou et al., 2011; Lu et al., 2018). Recent research illustrated the effect of vibration on the YAP, a transcription factor that was significant to MSC osteogenesis. Thompson et al. (2020) discovered that the application of vibration increased the YAP nuclear shuttling and restored the basal nuclear levels of YAP, which led to MSC osteogenesis. In addition to differentiation, MSC migration is also regulated by vibration. Wei et al. (2016) discovered that the SDF-1/CXCR4 pathway enhanced the MSC migration in response to the vibration which promoted fracture healing.

#### 2.2.5 Low Intensity Pulsed Ultrasound

Besides stretching, compression, fluid shear stress and vibration, LIPUS is also found to be a type of force to promote bone formation (Uddin and Qin, 2013). Gao et al. (2016) discussed the distinct pathways of MSCs from different sources in LIPUS-stimulated proliferation. LIPUS increased all MSC types proliferation. ERK1/2 was activated in dental pulp stem cells (DPSCs) and JNK MAPK signaling was activated in BMSCs after LIPUS application. However, in PDLSCs, JNK MAPK signaling was stimulated immediately after the application of LIPUS and p-p38 MAPK was increased subsequently. In spite of proliferation, LIPUS also promotes the MSCs migration in bone healing possibly through activating the SDF-1/CXCR4 signaling (Wei et al., 2014). In addition to the proliferation and migration, several studies illustrated that LIPUS led to a better osteointegration (Hui et al., 2011). The possibly osteogenic differentiation mechanism is activating of Rho-associated kinase-Cot/Tpl2-MEK-ERK signaling pathway (Kusuyama et al., 2014). However, the effectiveness of LIPUS in osteogenesis is open to debate. A recent study suggested that according to multiple randomized controlled trials in clinic, LIPUS possibly has no effect on radiographic bone healing (Schandelmaier et al., 2017).

## 3 Mechanical Stimulation Regulates Mesenchymal Stem Cell Surrounding Microenvironments in Bone Regeneration

**FIGURE 2:**
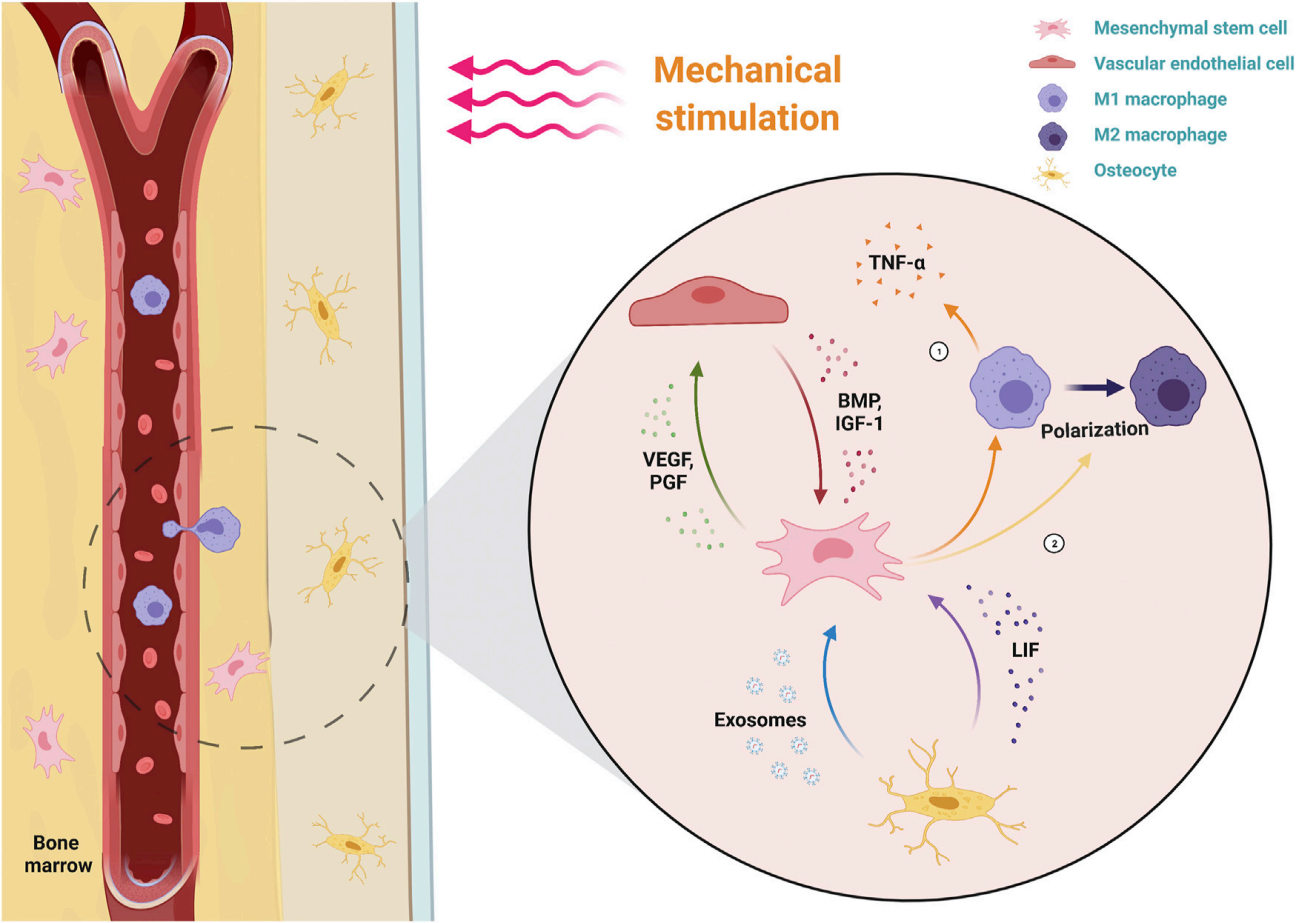
Dynamic interactions of MSCs with their microenvironment under mechanical stimulation. MSCs perceive the mechanical stimulation applied to the bone, which downregulates the inflammatory response by decreasing macrophage secretion of pro-inflammatory TNF-α and promoting the polarization of M1 macrophages (pro-inflammatory type) to M2 macrophages (anti-inflammatory type). Simultaneously, MSCs promote VEC angiogenesis by secreting angiogenic factors (VEGF, PGF). Osteogenic factors (BMP, IGF-1) secreted by mechanical activated-VECs, as well as LIF and exosomes secreted by mechanical activated-osteocytes, together promote the osteogenic differentiation of MSCs. TNF-α, tumor necrosis factor alpha; VECs, vascular endothelial cells; VEGF, vascular endothelial growth factor; PlGF, placental growth factor; BMP, bone morphogenetic protein; IGF-1, insulin-like growth factor 1; LIF, leukemia inhibitory factor.

Mscniches provide a microenvironment to support MSC self-renewal and multi-lineage differentiation. Bone regeneration involves the inflammatory responses of immune cells, blood vessel formation of endothelial cells and osteogenic process of MSCs. Thus, intercellular communication within the niche is crucial for bone regeneration and investigating the crosstalk between MSCs and other cells, including macrophages, vascular endothelial cells and osteocytes. As the crucial components of bone regeneration, blood vessel formation and inflammation are regulated by mechanical stimulation (Charoenpanich et al., 2014). Thus, it is important to ascertain the role of mechanical stimulation in the crosstalk of the osteogenic process (Figure 2).

### 3.1 Mechanical Stimulation Regulates the Immunoenvironment by Regulating the Crosstalk Between Mesenchymal Stem Cells and Macrophages

Some immune-inflammatory diseases, such as arthritis, suggest a correlation between mechanical force and the inflammatory response. Arthritis is characterized by inflammation that localized to the joints (such as the knee joint) when exposed to prolonged mechanical force. Therefore, mechanical force may be a factor that determines the transition of inflammation from systemic autoimmunity to local inflammation. According to Cambré’s research, MSCs in the mechanosensitive region of joints could sense mechanical stimulation and convert mechanical signals into chemical signals to trigger local inflammation and bone destruction, which ultimately led to the occurrence of arthritis (Cambre et al., 2018). Therefore, mechanical stimulation plays an essential role in inflammatory response (Hao et al., 2015).

Bone regeneration involves multiple stages and cell interactions. The formation of fracture hematoma and the subsequent acute inflammatory phase are key steps to determine the success of bone regeneration. The acute inflammatory phase begins with the activation of neutrophils that secrete inflammatory factors and chemokines to recruit monocytes and macrophages (Xing et al., 2010). In addition to cleaning up the necrotic tissue, macrophages secrete inflammatory cytokines and chemokines (such as TNF-α, IL-1β, IL-6, and CCL2) to recruit MSCs. Subsequently, MSC-rich granulation tissue replaces the hematoma. Immediately after, MSCs are stimulated by various factors in the environment to trigger osteogenic differentiation by either endochondral ossification or intramembranous ossification. Therefore, it is evident that an appropriate acute inflammatory phase duration is important for bone regeneration. The interactions between MSCs and macrophages dynamically regulate this phase. Macrophages have two phenotypes. The first is classically activated M1 macrophages, which function in initiating and sustaining inflammation, and the second is alternatively activated M2 macrophages, which function in resolving inflammation. The transformation of macrophages from M1 to M2 is known as macrophage polarization (Pugin et al., 1998). The main method of anti-inflammation in bone regeneration at this stage is the early polarization of pro-inflammatory M1 macrophages to anti-inflammatory M2 macrophages, which serves to promote the resolution of inflammation and the osteogenesis process.

Following the occurrence of a fracture, MSCs are mobilized into the peripheral blood by transforming growth factor-beta (TGF-β) (Wan et al., 2012). MSCs are exposed to fluid shear stresses, one of which is wall shear stress (WSS). WSS can stimulate MSCs to produce antioxidant and anti-inflammatory mediators. Additionally, the application of WSS to MSCs facilitates the recruitment of chemokines, including prostaglandin E2 (PEG2) and cyclooxygenase-2 (COX2), to inhibit the synthesis of tumor necrosis factor α (TNF-α) generated by immune cells and the inflammatory response (Diaz et al., 2017). The mechanism by which WSS stimulates MSCs to produce inflammatory mediators may be related to the FAK-COX2 signaling pathway. Lee et al.’s (2017) study showed that WSS could promote Ca^2+^ release and activate the Akt, MAPK and FAK signaling pathways of MSCs. When inhibited the above factors respectively, only FAK disrupted the induction of COX2 and decreased the production of MSC inflammatory mediators. Thus, the FAK-COX2 signaling pathway is significant for MSCs to respond to mechanical stimulation for immunomodulatory functions. In addition to shear stress, dynamic compression regulates the crosstalk between MSCs and macrophages. Zhang et al. (2021) developed an extracellular matrix-based hydroxyapatite scaffold fabricated by freeze-drying the ECM of compression-stimulated MSCs. This biofabricated scaffold could accelerate the polarization of macrophages from the pro-inflammatory M1 phenotype to the anti-inflammatory M2 phenotype to promote bone regeneration. These findings suggested that compression could promote the secretion of anti-inflammatory mediators in MSCs. However, recent studies have found that MSCs maintained their physiological levels through TNF-α endocytosis. Cyclic stretching promoted the proliferation and osteogenic differentiation of MSCs by TNFα endocytosis, which downregulated TNFα secretion in MSCs, rather than directly downregulating TNFα gene expression (Yu et al., 2021). Additionally, mechanical stimulation of adipose tissue modulates the anti-inflammatory properties of human adipose-derived stem cells (hADSCs) in adipose tissue. Carelli et al. compared the anti-inflammatory properties of hADSCs in mechanically stimulated adipose tissue and the control group. It was found that the anti-inflammatory effect of mechanical stimulated-hADSCs was superior to that of the control group hADSCs (Carelli et al., 2018). However, other studies have found that mechanical stimulation could promote inflammation and osteogenesis simultaneously, likely as a result of the MSC autocrine regulation of inflammatory factor secretion (Sumanasinghe et al., 2009; He et al., 2020).

Most studies have discussed the response of MSCs to mechanical stimulation during osteogenesis. A recent research found that macrophages were also capable of responding to mechanical stimulation (Dong et al., 2021). Mechanical stretch polarizes macrophages into the M2 phenotype that secrets inflammation-related cytokines, including IL10 and TGF-β, to regulate the local inflammatory microenvironment. Mechanical stimulation activates the YAP/BMP2 axis in macrophages to increase the expression of BMP2, which promotes the osteogenesis of MSCs. As an important component of the mechanical transduction pathway, YAP induces the polarization of M2 macrophages *via* Wnt5a and TGFβ1 (Feng et al., 2018). Schoenenberger et al. (2020) found that macrophages, as mechanosensitive cells, played an essential role in tendon repair. Mechanical stimulation was found to promote the transformation of macrophages to the M2 phenotype and subsequent tissue healing. These results suggested that future consideration might be given to exploring the role of mechanical stimulation in MSC and macrophage co-culture models.

### 3.2 Mechanical Stimulation Regulates the Angiogenic Microenvironment by Regulating the Crosstalk Between Mesenchymal Stem Cells and Vascular Endothelial Cells

Bone regeneration contains endochondral ossification and intramembranous ossification. Endochondral ossification is the process that stable cartilaginous soft callus first formed, followed by the formation of bone hard callus through vascular ingrowth and ossification centers (Gerstenfeld et al., 2003). The intramembranous osteogenesis process is accomplished by the differentiation of MSCs into osteoblasts at vascular-rich sites and the mineralization of osteoblasts to osteocytes. Thus, vascular formation, which is closely related to the osteogenesis process, is an important part of bone regeneration. During bone regeneration, MSCs and vascular endothelial cells (VECs) communicate with each other through paracrine mediators to promote osteogenesis (Li C.-J. et al., 2015). Mechanical stimulation is one of the biophysical factors that promote osteogenesis, and plays a crucial role in the crosstalk between MSCs and VECs.

Vascular endothelial growth factor (VEGF), a widely known signaling molecule regulating osteogenesis and vascularization, has been identified for its role in mechanical stimulation-induced osteogenesis. Charoenpanich et al. evaluated the effect of stretching on human MSC gene expression by microarray analysis. The results showed that stretching promoted the release of VEGF from human MSCs (Charoenpanich et al., 2014). Moreover, Jiang’s team found that stretching-stimulated VEGF secretion of MSCs not only promoted tube formation but also promoted VECs to release growth factors associated with bone formation, such as BMP-2 and IGF-1, which in turn regulated the osteogenesis process of MSCs (Jiang et al., 2018). In addition to stretching, dynamic compression can promote increased VEGF secretion in MSCs. Dynamic compression promotes VEGF secretion by upregulating YAP signaling activity in MSCs (Bandaru et al., 2020). In addition to VEGF, the expression level of placental growth factor (PlGF) in MSCs is related to the magnitude and duration of mechanical stimulation. PlGF has a variety of functions, including promoting osteogenesis and angiogenesis, and plays an essential role in the regulation of osteogenic-angiogenic interactions by mechanical stimulation (McCoy et al., 2013). It has also been found that mechanical stimulation can stimulate H vessel formation and VEGF secretion by downregulating exosomal miR-214-3p from MSCs (Wang et al., 2021).

In addition to the above *in vitro* studies, several *in vivo* experiments have investigated the effects of mechanical stimulation on bone regeneration and vascularization. Some studies have explored the effect of the initial application time of mechanical stimulation on vessel and bone formation. Boerckel et al., 2011 found that the application of mechanical loading in the early stage of bone defects could inhibit the growth of blood vessels into the defect area and lead to the failure of bone regeneration. In contrast, the application of mechanical loading delayed for 4 weeks could promote the reconstruction of blood vessel networks and bone regeneration (Boerckel et al., 2011). This result suggested that the effect of mechanical stimulation on vascularization and bone formation depended on the initial application time. McDermott et al. (2019) speculated that the difference was due to the different origin of the vessel forming at different times. Other studies have explored the effect of the loading application mode on revascularization. Claes et al., 2018 compared the effects of compression, stretching and shear stress on the vessel density in bone regeneration. As a result, the vessel density in the compression group was significantly higher than that in the other two groups, which suggested that compression was more beneficial to the bone regeneration process.

### 3.3 Mechanical Stimulation Regulates the Osteogenic Microenvironment by Regulating the Crosstalk Between Mesenchymal Stem Cells and Osteocytes

Osteocytes are mechanosensitive cells that reside in the lacunar-canalicular system (LCS) of cortical bone (Timmins and Wall, 1977). Recent studies have found several critical mechanical sensors of osteocytes, such as cilia, integrin, ion channels and G-protein-coupled receptors (Uda et al., 2017). Osteocytes regulate bone remodeling, mainly by sensing fluid shear stress caused by mechanical loading and regulating osteoblast-osteoclast communication (Dallas et al., 2013). As mentioned above, osteocytes play an important role in responding to mechanical stimulation. Osteocytes function as regulators influencing bone loss and formation by modulating osteoblast-osteoclast coupling. Osteocytes are of vital importance in the reconstruction of bone defects (Robling and Bonewald, 2020). Osteocytes regulate bone regeneration in both direct and indirect ways: secreting stimulators and inhibitors that affect osteoblast activity, and modulating osteoclast activity to regulate osteoblast behavior indirectly (Robling and Bonewald, 2020). However, due to limited research methods, the role of the osteocyte response to mechanical stimulation in bone regeneration has not been fully explored.

Osteocytes respond to external mechanical stimulation by secreting soluble factors that regulate MSC gene expression. Specific communications exist between osteoblasts and MSCs under mechanical stimulation conditions. (Hoey et al., 2011) found that conditional medium for mechanical stimulation of osteocytes upregulated osteogenic gene expression in MSCs, while no upregulation was seen in osteoblasts treated with the same. This suggested that mechanical stimulation played a vital role in the crosstalk between osteocytes and MSCs. Several studies have explored the paracrine mechanism by which osteocytes regulated MSC osteogenesis in response to mechanical stimulation. Du J. et al. (2020) suggested that mechanical regulating osteoblast-osteoclast coupling by promoting osteocyte secretion of leukemia inhibitory factor (LIF). Extracellular vesicles (EVs), as specific components of cell-cell and cell-matrix communication, also play an important role in osteocyte and MSC interactions under mechanical stimulation. Eichholz et al. (2020) comprehensively characterized the proteins secreted by osteoblasts after fluid shear stress through proteomics and found that proteins associated with EVs were significantly overexpressed. Moreover, culturing MSCs with the collected EVs resulted in MSC osteogenic differentiation, suggesting that mechanical stimulation promotes osteocytes to modulate MSC behavior *via* EVs. Peiying Lv’s team found that exosomes produced by osteocytes following mechanical stimulation also promoted the osteogenic differentiation of human periodontal ligament stem cells (PDLSC) (Lv et al., 2020).

## 4 Applications of Mechanical Stimulation to Mesenchymal Stem Cells in Bone Regeneration

Different forms of mechanical force have been described previously to promote osteogenic differentiation of MSCs. However, these studies did not discuss the optimal mode of application of mechanical stimulation in detail. Therefore, several mechanical application modes will be discussed in this part, including the magnitude, frequency, intermittent or continuous, immediate or delayed application, and the dimensionalities of mechanical stimulation (Table 1).

**TABLE 1:** Applications of mechanical stimulation on MSCs in bone regeneration.

Source of MSC	Force type	Mechanical parameter	Intermittent and continuous loading	Immediate or delayed loading	Dimensionality	Discoveries	References
*Mus musculus*	Shear stress	1, 2, 5 Pa; 0.5, 1, 2 Hz	Intermittent: 1, 2, 4 h/day	1–3 days	2D	2 Pa and 2 Hz has a superior osteogenic effect	Stavenschi et al. (2017)
*Rattus norregicus*	Shear stress	1.03, 0.1, 0.01, 0.001 Pa	1) Continuous	40 h	2D	Intermittent loading for 0.01 Pa has a superior osteogenic effect	Dash et al. (2020)
			2) Intermittent: application 1 h + Intermittent 7 h				
*Homo sapiens*	Shear stress	0.01 Pa	Continuous	24 h	3D (borosilicate glass capillary tubes)	Loading regime of 0.01Pa has a superior osteogenic effect	Xue and Cartmell, (2020)
*Homo sapiens*	Shear stress	0.005, 0.011, 0.015 Pa; 3, 6, 9 ml/min	Continuous	24 h	3D (porous cylindrical β-TCP scaffold)	15 mPa has a superior osteogenic effect	Li et al. (2009)
*Homo sapiens*	Shear stress	0.34 Pa (0.3 ml/min), 0.42 Pa (4 ml/min)	1) Continuous: 0.42 Pa (4 ml/min)	4 h	3D [porous poly lactic co-glycol acid (PLGA)]	Intermittent FSS has a superior osteogenic effect	Liu et al. (2012)
			2) Intermittent: 0.42 Pa (4 ml/min) 1 h + 0.34 Pa (0.3 ml/min) 11 h				
Mus musculus	Tensile	10% Elongation; 0.5 Hz	Intermittent: 12 h/day	48–72 h	2D	CMS has a superior osteogenic effect	Wang et al. (2017)
*Homo sapiens*	Tensile	10% Elongation; 0.1%/s	Intermittent: 2 h/day	—	3D (PCL nanofibrous scaffolds)	10% Elongation enhances long-term ECM deposition and differentiation	Nathan et al. (2011)
*Bos taurus*	Tensile	1) Continuous: 10% elongation; 2.5%/min	1) Continuous: 2 h	48 h	3D (PCL nanofibrous scaffolds)	Elongation stiffened and condensed MSC nuclei	Heo et al. (2016)
		2) Intermittent: 3% elongation; 1 Hz	2) Intermittent: 6 h/day				
*Homo sapiens*	Tensile	10% Elongation; 0.5 Hz	Continuous	—	2D	10% Elongation has a superior osteogenic effect	Fang et al. (2019)
						Tensile inhibited adipogenesis, but promoted osteogenesis	
*Homo sapiens*	Compression	0.22% strain; 1 Hz	Intermittent: 4 h/day	24 h	3D (PCL-TCP scaffold)	0.22% compressive strain has a superior osteogenic effect	Ravichandran et al. (2017)
*Homo sapiens*	Compression	1) 10% Elongation; 1 Hz	Intermittent: 4 h/day	-	3D (collagen–alginate scaffolds)	10% compressive strain has a superior osteogenic effect	Michalopoulos et al. (2012)
		2) 15% strain; 1 Hz				15% cyclic compressive strain has a superior chondrogenic effect	
*Oryctolagus cuniculus*	Compression	10% strain; 1 Hz	Intermittent: 2 h/day	-	3D (collagen scaffold)	0.22% compressive strain has a superior chondrogenic effect	Cao et al. (2019)
*Homo sapiens*	Compression	0.06–0.94 mPa; 1 Hz	Intermittent: 15 min/day	48 h	3D (hydroxyapatite scaffolds)	0.06–0.94 mPa compressive strain has a superior chondrogenic effect. And can modulating the inflammatory microenvironment	Zhang et al. (2021)

### 4.1 Magnitude and Frequency of Mechanical Stimulation

The osteogenic differentiation of MSCs has been found to be correlated with the magnitude and frequency of mechanical stimulation. Stavenschi et al. explored the osteogenic effect of oscillatory fluid flow on MSCs of 1 Pa, 2 and 5 Pa. The results showed that the expression of osteogenic genes was significantly upregulated at the magnitude of 2 Pa and the frequency of 2 Hz (Stavenschi et al., 2017). The most effective shear stress for promoting MSC proliferation and osteogenesis has also been explored. Sanat’s research showed that MSCs exhibited a high cell proliferation rate when stimulated by intermittent flow at 1.09 mPa, while 10 mPa upregulated osteogenic gene expression (Dash et al., 2020). Xue and Cartmell, (2020) suggested the osteogenic effect of shear stress on MSCs in three-dimensional culture was different from that in the plate. Lower fluid shear stress (1–10 mPa) stimulated MSCs in the scaffold used to simulate a three-dimensional environment to promote the osteogenic differentiation, whereas 100–4,000 mPa was required when the MSCs were cultured in a plate (Xue and Cartmell, 2020).

### 4.2 Intermittent and Continuous Mechanical Stimulation

Recent studies have shown that, compared to long-term continuous mechanical stimulation, a period of rest time during mechanical stimulation enhanced bone formation and improved the mechanical properties of bone (Robling et al., 2002; Saxon et al., 2005). Compared to continuous shear stress, intermittent application has been proven to maintain the mechanosensitivity of MSCs and osteocytes (Siller-Jackson et al., 2008; Liu et al., 2012). In addition to FSS, intermittent stretching can promote osteogenic differentiation of MSCs (Wang et al., 2017). Continuous cyclic mechanical tension (CCMT) has been found to downregulate Runx2 expression in MSCs and inhibit osteogenic differentiation (Shi et al., 2011). Another study compared the effects of intermittent compressive force (ICF) and continuous compressive force (CCF) on the behavior of PDLSCs. The results suggested that ICF upregulated TGFβ-1 and promoted the osteogenic differentiation of PDLSCs, whereas the osteogenic gene expression of the CCF group was unchanged (Manokawinchoke et al., 2019). Therefore, the intermittent mechanical stimulation mode is superior to continuous mechanical stimulation in terms of promoting bone regeneration.

At present, intermittent mechanical stimulation promotes osteogenesis in bone that needs the loading interval to recover its mechano-sensitivity to mechanical signals. Nardone et al. (2017) found that mechano-sensing switches (such as pFAK) were released from FAs into the cytoplasm during intermittent and activated YAP. This revocation of activation suggested that intermittent mechanical loading could activate integrin signaling downstream, which possibly explained the decreased mechano-sensitivity of bone tissue caused by continuous mechanical stimulation. Additionally, the mechanical environment is capable of modulating nuclear properties, and mechanical sensitivity may also be related to the nuclear biophysical properties (Heo et al., 2016). The nuclei in dynamic loading induced MSCs to stiffen and become resistant to deformation, which sensitizes MSCs to mechanical stimulated calcium signaling and differentiated marker expression (Heo et al., 2016). Thus, the nucleus plays an essential role in modulating cellular mechano-sensation during differentiation. There is limited research on the mechanisms by which MSCs respond to intermittent and continuous stimulation. However, studies on the mechanism of osteoblasts could provide hints for future research. It was suggested that mechano-sensitivity is primarily associated with actin stress fibers. Gardinier et al. suggested that osteoblasts responded to FSS through actin stress fiber formation (ASFF), and ASFF led to increased cell stiffness and decreased mechano-sensitivity (Gardinier et al., 2014). LIM kinase 2 (LIMK2) is a gene related to the reorganization of the cytoskeleton. Several studies found that inhibiting the LIMK2 increased the sensitivity of ERK1/2 to fluid shear stress and promoted the gene expression of c-fos to enhance the mechanical sensitivity of osteoblasts (Zhang et al., 2009; Xiang et al., 2012). These results suggested that the mechanism by which MSCs respond to intermittent mechanical stimulation may also be related to the actin stress fiber and cytoskeleton.

### 4.3 Immediate and Delayed Mechanical Stimulation

Delayed mechanical stimulation has a positive effect on osteogenesis by promoting angiogenesis. The formation of blood vessels is closely related to endochondral ossification, in which MSCs first aggregate and differentiate into hyaline cartilage to form the cartilage model. Following growth of blood vessels, the cartilage is gradually replaced by bone tissue. Joe’s laboratory investigated the effects of immediate and delayed mechanical loading on vascular ingrowth in bone regeneration (Boerckel et al., 2011; Ruehle et al., 2020). The results showed that load initiation was a key determinant of vascular network formation. Immediate loading significantly inhibited the growth of blood vessels into the bone defect area, which led to fracture nonunion. In contrast, delayed loading allowed the growth of vessels into the defect and induced vascular remodeling. The study by Anna showed that the bone accumulation rate was significantly elevated by 4-week delayed mechanical loading application, which coincided with chondrocyte hypertrophy and endochondral transition (McDermott et al., 2019). They concluded that a 4-week delay in mechanical loading better mimicked the process of endochondral ossification.

### 4.4 Dimensionalities of Mechanical Stimulation

The mechanical stimulation applied to cells in two-dimensional (2D) environments is unidirectional. However, mechanical stimulation is multidirectional in physiological environments. The behavior of mechanical loading-induced cells is altered by the dimensionalities of their environments. Thus, the response of cells to external mechanical forces in three-dimensional (3D) environments that mimic the physiological environments *in vivo* needs to be explored. Li and his team found that long-term compression loading induced maturation of α5-integrin-based adhesions to form 3D-matrix adhesions (3DMAs) in the 3D environment (Li et al., 2020). In contrast to the FA formed in the 2D environment, the composition and morphology of 3DMAs are found only in native tissues and cell-derived matrices, suggesting that dimensionality influences the behavior of cells under mechanical stimulation. However, the exact mechanism by which external mechanical forces regulate cell fate in different dimensionalities remains unclear.

Exploring the effects of mechanical stimulation on cells in a 3D environment involves not only mechanical stimulation of cells but also the properties of materials. In 2D conditions, mechanical stimulation is applied to the cells directly. However, in a 3D environment, the force is first applied to the substrate which then transmits the mechanical signals to the cell through the deformation generated by the stimulation (Steinmetz et al., 2015). Thus, the process by which the mechanical signals are transmitted to cells contains two steps: the deformation of scaffolds produced by mechanical stimulation and the cell sensing and responding to the deformation. The ECM is not a linearly elastic material and has complex mechanical properties, including viscoelasticity, mechanical plasticity and nonlinear elasticity (Chaudhuri et al., 2020). The ECM responds to external mechanical stimulation by remodeling the stress fiber network, such as by changing the structure of the fiber network and forming bonds between the fibers (Loebel et al., 2019). The effect of mechanical stimulation on cells is related to the interactions between ECM properties and cells, which suggests that the mechanical properties of scaffold materials are of vital importance for cell differentiation in the 3D environment.

Materials that mimic the mechanical properties of ECM have been explored. Davidson and his team developed a multifiber hydrogel network with force-responsive characteristics (Davidson et al., 2020). In this network, the fibers form covalent bonds under mechanical loading, and the interactions of the fiber increase material stiffness and plastic deformation. Davidson’s design mimics the physiological process of ECM remodeling under mechanical stimulation, providing a model for exploring the effects of mechanical stimulation on cells in 3D environments (Davidson et al., 2020). Mechanical stimulation in a 3D environment fabricates the layered scaffolds with gradient mechanical properties. Horner et al. (2019) designed a 3D electrospinning scaffold with a tissue gradient that generates spatially controlled strain gradients in a scaffold depth-dependent manner under dynamic loading. MSCs in the greater compressive strain areas upregulate osteogenic gene expression, while chondrogenic markers are upregulated in the high local compressive strain areas. The formation of the mechanical gradient was maintained only under the application of dynamic loading. This study shows that regulating the local mechanical microenvironment provides a strategy to recapitulate the gradient structure of osteochondral tissues (Table 1).

Explorations of optimal mechanical parameters are significant for further application of mechanical stimulation in bone tissue engineering as presented above. Shear stress from 1.09 mPa to 5 Pa was applied to MSCs in previous studies, and 10–15 mPa were proved to have a superior osteogenic effect. Stretching or compression resulted 10% strain was discovered promoting osteogenesis. In comparison with continuous mechanical stimulation, the intermittent application is more efficient in inducing osteogenic differentiation *via* maintaining the mechanosensitivity of MSCs to mechanical signals. Therefore, mechanical stimulations are recommended to be performed with appropriate intervals. Application of delayed mechanical stimulation was reported to be an ideal option for facilitating angiogenesis in bone remodeling, which indicates that future researches should take the mechanical stimulation application time into consideration. Besides, in contrast to the 2D environment, MSCs showed a more bionic behavior in response to external mechanical stimulation in 3D environment that mimics physiological environments. Thus, 3D environment is recommended for the mechanical stimulation application.

## 5 Outlook

Mechanical stimulation plays an important role in bone regeneration due to its influences on bone physiological functions. The main functional cells in bone regeneration, BMSCs, sense specific mechanical signals through mechanosensors on the cytomembrane, which results in the activation of downstream molecular pathways and altered expression of osteogenic genes. Mechanical stimulated-MSCs regulate immune, angiogenic and osteogenic microenvironments of bone regeneration by interacting with macrophages, endothelial cells and osteocytes. Modes of mechanical stimulation including the magnitude, frequency, duration and intermittence, affect the osteogenic differentiation of MSCs. Therefore, investigations of mechanical stimulation on bone regeneration for application in regenerative medicine are of great importance.

The mechanism of mechanical stimulation for osteogenesis has been studied in two main aspects, the principle of mechanosensors on the cell membrane surface to sense mechanical stimulation and the intracellular pathways transmitting mechanical signals, which ultimately lead to changes in gene expression. The mechanism of the mechanoreceptor, including integrated proteins and primary cilia, has been widely reported (Hoey et al., 2012; Kechagia et al., 2019). However, as a recently discovered mechanosensitive calcium ion channel, the principle of the Piezo1 response to mechanical stimulation has not been fully elucidated in MSCs. Therefore, the mechanism of PIEZO1 responding to mechanical stimulation in MSCs needs to be confirmed by more researchers to provide convincing evidence for future applications in bone regeneration. Additionally, in spite of advancements in exploring mechanotransduction in 2D environment, our knowledge of the MSC behaviors in 3D environments under mechanical stimulation remains limited. 3D culture is one of the necessary factors for the construction of the tissue engineered-bone which mimics physiological environments and provides more suitable matrix for MSCs. The mechanism of MSCs responding to mechanical stimuli in 3D environments is possibly the priority for future researches.

The effect of mechanical stimulation on the cross-talk between MSCs and osteogenesis-related cells is an emerging field of vital significance for bone regeneration. 1) Osteocytes are mechanosensitive cells that resided in the mineral matrix, which play an important role in modulating bone metabolism (Timmins and Wall, 1977). And the interactions between MSCs and osteocytes under external mechanical stimulation deserve further investigation, especially the means by which paracrine regulation of the loading induced-osteocytes regulates the behavior of MSCs. Investigating the interactions between osteocytes and MSCs under mechanical stimulation contributes to a better understanding of MSC response to mechanical stimulation and the comprehensive effect of mechanical stimulation on bone. 2) Excesses of inflammatory response often result in the failure of bone repair in bone tissue engineering. A few studies have illustrated that mechanical stimulation could facilitate the resolution of inflammation through regulating the interactions between MSCs and macrophages. However, the anti-inflammatory mechanism and the optimal application paraments remain unclear. Thus, further studies on the role of mechanical stimulation in the immune microenvironment during bone regeneration may provide a new insight into the design of bone regeneration biomaterials. 3) Interactions between endothelial cells and MSCs under mechanical stimulation also attract great attention. Studies proved that delayed mechanical stimulation promotes angiogenesis in bone regeneration. However, most of the studies only adopted a single delayed time point and the temporal effect of different delayed-loading time points is not clear. Therefore, studies on the effect of mechanical stimulation loading time in interactions between MSCs and endothelial cells can provide a comprehensive understanding of angiogenesis, which further guides the weight-bearing point of the fracture patients.

Mechanical stimulation has been used as a therapy in orthopedic which is known as mechanotherapy (Huang et al., 2013). For instance, distraction osteogenesis is used to correct limb and craniofacial defects, and LIPUS is used to hasten the fracture healing process and increase bone mass. However, current approaches are applying mechanical stimulation directly to the tissue, rather than through the substrate. However, efficiencies of these mechanotherapies in bone repairing are open to debate, as a recent systematic review concluded that LIPUS did not improve outcomes important to patients (Schandelmaier et al., 2017). The potential application may combine mechanical stimulation and bone tissue engineering. As the key element of bone tissue engineering, 3D culture involves the interactions between the cells and the materials. Scientists are keeping searching for materials that are more compatible with physiological deformation, retraction and osteogenic activity in mechanical environments. And the interactions between cells and biomaterials also require continuous refinement, further work may focus on the combined effect of the substance stiffness and the external mechanical stimulation application on MSCs. Several active biomaterials offer novel approaches to apply mechanical stimulation, such as magnetically triggered systems. Due to the variable mechanical parameters and the precise controlling of the mechanical application timepoint, magnetically triggered strategies will possibly receive increasing attention.

## 6 Conclusion

External mechanical force plays an essential role in bone regeneration. And MSCs can sense and respond to mechanical signals during this process. Thus, in this review we discussed MSCs mechanotransduction mechanisms, the influences of mechanical stimulation on modulating interactions between MSCs and surrounding cells in bone regeneration including the immune, angiogenic and osteogenic microenvironments, and the applications of mechanical stimulation of MSCs in bone regeneration. The description of MSCs mechanotransduction on purpose of providing a comprehensive view and several promising mechanosensors required to be fully investigated in MSC mechanotransduction field. The regulation of mechanical stimulation on microenvironments surrounding MSC discussed in the manuscript is of great significance for the bone regenerative medicine, which offers an insight for the design of tissue engineered bone in consideration of immune response, angiogenesis and osteogenesis. Moreover, the depiction of different mechanical stimulation application modes bring insightful guidance to the design of bone regenerative biomaterials and clinical applications of the mechanical stimulation.

## References

[B1] Argentati C. Morena F. Tortorella I. Bazzucchi M. Porcellati S. Emiliani C. (2019). Insight into Mechanobiology: How Stem Cells Feel Mechanical Forces and Orchestrate Biological Functions. Ijms 20 (21), 5337. 10.3390/ijms20215337 PMC6862138 31717803

[B2] Baas E. Kuiper J. H. Yang Y. Wood M. A. El Haj A. J. (2010). *In Vitro* bone Growth Responds to Local Mechanical Strain in Three-Dimensional Polymer Scaffolds. J. Biomech. 43 (4), 733–739. 10.1016/j.jbiomech.2009.10.016 19942222

[B3] Bandaru P. Cefaloni G. Vajhadin F. Lee K. Kim H. J. Cho H. J. (2020). Mechanical Cues Regulating Proangiogenic Potential of Human Mesenchymal Stem Cells through YAP‐Mediated Mechanosensing. Small 16 (25), 2001837. 10.1002/smll.202001837 PMC7523466 32419312

[B4] Boerckel J. D. Uhrig B. A. Willett N. J. Huebsch N. Guldberg R. E. (2011). Mechanical Regulation of Vascular Growth and Tissue Regeneration *In Vivo* . Proc. Natl. Acad. Sci. 108 (37), E674–E680. 10.1073/pnas.1107019108 21876139 PMC3174614

[B5] Bouzid T. Kim E. Riehl B. D. Esfahani A. M. Rosenbohm J. Yang R. (2019). The LINC Complex, Mechanotransduction, and Mesenchymal Stem Cell Function and Fate. J. Biol. Eng. 13, 68. 10.1186/s13036-019-0197-9 31406505 PMC6686368

[B6] Cambré I. Gaublomme D. Burssens A. Jacques P. Schryvers N. De Muynck A. (2018). Mechanical Strain Determines the Site-specific Localization of Inflammation and Tissue Damage in Arthritis. Nat. Commun. 9 (1), 4613. 10.1038/s41467-018-06933-4 30397205 PMC6218475

[B7] Cao W. Lin W. Cai H. Chen Y. Man Y. Liang J. (2019). Dynamic Mechanical Loading Facilitated Chondrogenic Differentiation of Rabbit BMSCs in Collagen Scaffolds. Regen. Biomater. 6 (2), 99–106. 10.1093/rb/rbz005 30967964 PMC6446999

[B8] Carelli S. Colli M. Vinci V. Caviggioli F. Klinger M. Gorio A. (2018). Mechanical Activation of Adipose Tissue and Derived Mesenchymal Stem Cells: Novel Anti-inflammatory Properties. Ijms 19 (1), 267. 10.3390/ijms19010267 PMC5796213 29337886

[B9] Charoenpanich A. Wall M. E. Tucker C. J. Andrews D. M. K. Lalush D. S. Dirschl D. R. (2014). Cyclic Tensile Strain Enhances Osteogenesis and Angiogenesis in Mesenchymal Stem Cells from Osteoporotic Donors. Tissue Eng. A 20 (1-2), 67–78. 10.1089/ten.TEA.2013.0006 PMC3875187 23927731

[B10] Chaudhuri O. Cooper-White J. Janmey P. A. Mooney D. J. Shenoy V. B. (2020). Effects of Extracellular Matrix Viscoelasticity on Cellular Behaviour. Nature 584 (7822), 535–546. 10.1038/s41586-020-2612-2 32848221 PMC7676152

[B11] Chen B. Lin T. Yang X. Li Y. Xie D. Zheng W. (2016). Low-magnitude, High-Frequency Vibration Promotes the Adhesion and the Osteogenic Differentiation of Bone Marrow-Derived Mesenchymal Stem Cells Cultured on a Hydroxyapatite-Coated Surface: The Direct Role of Wnt/β-Catenin Signaling Pathway Activation. Int. J. Mol. Med. 38 (5), 1531–1540. 10.3892/ijmm.2016.2757 28026000

[B12] Chen N. X. Ryder K. D. Pavalko F. M. Turner C. H. Burr D. B. Qiu J. (2000). Ca2+ Regulates Fluid Shear-Induced Cytoskeletal Reorganization and Gene Expression in Osteoblasts. Am. J. Physiology-Cell Physiol. 278 (5), C989–C997. 10.1152/ajpcell.2000.278.5.C989 10794673

[B13] Chen S. Jing J. Yuan Y. Feng J. Han X. Wen Q. (2020). Runx2+ Niche Cells Maintain Incisor Mesenchymal Tissue Homeostasis through IGF Signaling. Cel Rep. 32 (6), 108007. 10.1016/j.celrep.2020.108007 PMC7461627 32783935

[B14] Chen X. He F. Zhong D.-Y. Luo Z.-P. (2015). Acoustic-frequency Vibratory Stimulation Regulates the Balance between Osteogenesis and Adipogenesis of Human Bone Marrow-Derived Mesenchymal Stem Cells. Biomed. Res. Int. 2015, 1–10. 10.1155/2015/540731 PMC4337172 25738155

[B15] Chen X. Yan J. He F. Zhong D. Yang H. Pei M. (2018). Mechanical Stretch Induces Antioxidant Responses and Osteogenic Differentiation in Human Mesenchymal Stem Cells through Activation of the AMPK-SIRT1 Signaling Pathway. Free Radic. Biol. Med. 126, 187–201. 10.1016/j.freeradbiomed.2018.08.001 30096433 PMC6165675

[B16] Ciani C. Sharma D. Doty S. B. Fritton S. P. (2014). Ovariectomy Enhances Mechanical Load-Induced Solute Transport Around Osteocytes in Rat Cancellous Bone. Bone 59, 229–234. 10.1016/j.bone.2013.11.026 24316418 PMC4358819

[B137] Claes L. Meyers N. Schülke J. Reitmaier S. Klose S. Ignatius A. (2018). The Mode of Interfragmentary Movement Affects Bone Formation and Revascularization After Callus Distraction. PLoS One 13, e0202702. 10.1371/journal.pone.0202702 30138362 PMC6107229

[B17] Corrigan M. A. Johnson G. P. Stavenschi E. Riffault M. Labour M.-N. Hoey D. A. (2018). TRPV4-mediates Oscillatory Fluid Shear Mechanotransduction in Mesenchymal Stem Cells in Part via the Primary Cilium. Sci. Rep. 8 (1), 3824. 10.1038/s41598-018-22174-3 29491434 PMC5830574

[B18] Coste B. Mathur J. Schmidt M. Earley T. J. Ranade S. Petrus M. J. (2010). Piezo1 and Piezo2 Are Essential Components of Distinct Mechanically Activated Cation Channels. Science 330 (6000), 55–60. 10.1126/science.1193270 20813920 PMC3062430

[B19] Dallas S. L. Prideaux M. Bonewald L. F. (2013). The Osteocyte: An Endocrine Cell … and More. Endocr. Rev. 34 (5), 658–690. 10.1210/er.2012-1026 23612223 PMC3785641

[B20] Dash S. K. Sharma V. Verma R. S. Das S. K. (2020). Low Intermittent Flow Promotes Rat Mesenchymal Stem Cell Differentiation in Logarithmic Fluid Shear Device. Biomicrofluidics 14 (5), 054107. 10.1063/5.0024437 33163135 PMC7595746

[B21] Davidson M. D. Ban E. Schoonen A. C. M. Lee M. H. D'Este M. Shenoy V. B. (2020). Mechanochemical Adhesion and Plasticity in Multifiber Hydrogel Networks. Adv. Mater. 32 (8), 1905719. 10.1002/adma.201905719 PMC7042082 31851400

[B22] Delaine-Smith R. M. Reilly G. C. (2011). The Effects of Mechanical Loading on Mesenchymal Stem Cell Differentiation and Matrix Production. Vitam Horm. 87, 417–480. 10.1016/b978-0-12-386015-6.00039-1 22127254

[B23] Dewey C. F. Jr. Bussolari S. R. Gimbrone M. A. Jr. Davies P. F. (1981). The Dynamic Response of Vascular Endothelial Cells to Fluid Shear Stress. J. Biomech. Eng. 103 (3), 177–185. 10.1115/1.3138276 7278196

[B24] Diaz M. F. Vaidya A. B. Evans S. M. Lee H. J. Aertker B. M. Alexander A. J. (2017). Biomechanical Forces Promote Immune Regulatory Function of Bone Marrow Mesenchymal Stromal Cells. Stem Cells 35 (5), 1259–1272. 10.1002/stem.2587 28181347 PMC5405000

[B25] Dong L. Song Y. Zhang Y. Zhao W. Wang C. Lin H. (2021). Mechanical Stretch Induces Osteogenesis through the Alternative Activation of Macrophages. J. Cel Physiol 236 (9), 6376–6390. 10.1002/jcp.30312 33634492

[B26] Du G. Li L. Zhang X. Liu J. Hao J. Zhu J. (2020a). Roles of TRPV4 and Piezo Channels in Stretch-Evoked Ca2+ Response in Chondrocytes. Exp. Biol. Med. (Maywood) 245 (3), 180–189. 10.1177/1535370219892601 31791130 PMC7045327

[B27] Du J. Yang J. He Z. Cui J. Yang Y. Xu M. (2020b). Osteoblast and Osteoclast Activity Affect Bone Remodeling upon Regulation by Mechanical Loading-Induced Leukemia Inhibitory Factor Expression in Osteocytes. Front. Mol. Biosci. 7, 585056. 10.3389/fmolb.2020.585056 33324677 PMC7726425

[B28] Duty A. O. Oest M. E. Guldberg R. E. (2007). Cyclic Mechanical Compression Increases Mineralization of Cell-Seeded Polymer Scaffolds *In Vivo* . J. Biomech. Eng. 129 (4), 531–539. 10.1115/1.2746375 17655474

[B29] Eichholz K. F. Woods I. Riffault M. Johnson G. P. Corrigan M. Lowry M. C. (2020). Human Bone Marrow Stem/stromal Cell Osteogenesis Is Regulated via Mechanically Activated Osteocyte-Derived Extracellular Vesicles. Stem Cell Transl Med 9 (11), 1431–1447. 10.1002/sctm.19-0405 PMC7581449 32672416

[B30] Einhorn T. A. Gerstenfeld L. C. (2015). Fracture Healing: Mechanisms and Interventions. Nat. Rev. Rheumatol. 11 (1), 45–54. 10.1038/nrrheum.2014.164 25266456 PMC4464690

[B31] Fang B. Liu Y. Zheng D. Shan S. Wang C. Gao Y. (2019). The Effects of Mechanical Stretch on the Biological Characteristics of Human Adipose‐derived Stem Cells. J. Cel Mol Med 23 (6), 4244–4255. 10.1111/jcmm.14314 PMC6533502 31020802

[B32] Federman M. Nichols G. Jr. (1974). Bone Cell Cilia: Vestigial or Functional Organelles? Calc. Tis Res. 17 (1), 81–85. 10.1007/bf02547216 4451879

[B33] Feng Y. Liang Y. Zhu X. Wang M. Gui Y. Lu Q. (2018). The Signaling Protein Wnt5a Promotes TGFβ1-Mediated Macrophage Polarization and Kidney Fibrosis by Inducing the Transcriptional Regulators Yap/Taz. J. Biol. Chem. 293 (50), 19290–19302. 10.1074/jbc.RA118.005457 30333225 PMC6302175

[B34] Fu J. Liu X. Tan L. Cui Z. Liang Y. Li Z. (2020). Modulation of the Mechanosensing of Mesenchymal Stem Cells by Laser-Induced Patterning for the Acceleration of Tissue Reconstruction through the Wnt/β-Catenin Signaling Pathway Activation. Acta Biomater. 101, 152–167. 10.1016/j.actbio.2019.10.041 31678738

[B35] Gao H. Zhai M. Wang P. Zhang X. Cai J. Chen X. (2017). Low-level Mechanical Vibration Enhances Osteoblastogenesis via a Canonical Wnt Signaling-Associated Mechanism. Mol. Med. Rep. 16 (1), 317–324. 10.3892/mmr.2017.6608 28534995

[B36] Gao Q. Walmsley A. D. Cooper P. R. Scheven B. A. (2016). Ultrasound Stimulation of Different Dental Stem Cell Populations: Role of Mitogen-Activated Protein Kinase Signaling. J. Endodontics 42 (3), 425–431. 10.1016/j.joen.2015.12.019 26830427

[B37] Gardinier J. Yang W. Madden G. R. Kronbergs A. Gangadharan V. Adams E. (2014). P2Y2 Receptors Regulate Osteoblast Mechanosensitivity during Fluid Flow. Am. J. Physiology-Cell Physiol. 306 (11), C1058–C1067. 10.1152/ajpcell.00254.2013 PMC4042092 24696143

[B38] Gerstenfeld L. C. Cullinane D. M. Barnes G. L. Graves D. T. Einhorn T. A. (2003). Fracture Healing as a post-natal Developmental Process: Molecular, Spatial, and Temporal Aspects of its Regulation. J. Cel. Biochem. 88 (5), 873–884. 10.1002/jcb.10435 12616527

[B39] Grier W. Moy A. S. Moy A. Harley B. (2017). Cyclic Tensile Strain Enhances Human Mesenchymal Stem Cell Smad 2/3 Activation and Tenogenic Differentiation in Anisotropic Collagen-Glycosaminoglycan Scaffolds. eCM 33, 227–239. 10.22203/eCM.v033a1410.22203/ecm.v033a17 28319248 PMC5453510

[B40] Gurkan U. A. Akkus O. (2008). The Mechanical Environment of Bone Marrow: a Review. Ann. Biomed. Eng. 36 (12), 1978–1991. 10.1007/s10439-008-9577-x 18855142

[B41] Hao J. Zhang Y. Jing D. Shen Y. Tang G. Huang S. (2015). Mechanobiology of Mesenchymal Stem Cells: Perspective into Mechanical Induction of MSC Fate. Acta Biomater. 20, 1–9. 10.1016/j.actbio.2015.04.008 25871537

[B42] He D. Liu F. Cui S. Jiang N. Yu H. Zhou Y. (2020). Mechanical Load-Induced H2S Production by Periodontal Ligament Stem Cells Activates M1 Macrophages to Promote Bone Remodeling and Tooth Movement via STAT1. Stem Cel Res Ther 11 (1), 112. 10.1186/s13287-020-01607-9 PMC7071778 32169104

[B43] Heo S.-J. Driscoll T. P. Thorpe S. D. Nerurkar N. L. Baker B. M. Yang M. T. (2016). Differentiation Alters Stem Cell Nuclear Architecture, Mechanics, and Mechano-Sensitivity. Elife 5. 10.7554/eLife.18207 PMC5148611 27901466

[B138] Hoey D. A. Kelly D. J. Jacobs C. R. (2011). A Role for the Primary Cilium in Paracrine Signaling Between Mechanically Stimulated Osteocytes and Mesenchymal Stem Cells. Biochem Biophys Res Commun. 412 (1), 182–187. 10.1016/j.bbrc.2011.07.072 21810408 PMC3160132

[B44] Hoey D. A. Tormey S. Ramcharan S. O'Brien F. J. Jacobs C. R. (2012). Primary Cilia‐Mediated Mechanotransduction in Human Mesenchymal Stem Cells. Stem Cells 30 (11), 2561–2570. 10.1002/stem.1235 22969057 PMC3533782

[B45] Holmes D. (2017). Non-union Bone Fracture: a Quicker Fix. Nature 550 (7677), S193. 10.1038/550S193a 29069072

[B46] Horner C. B. Hirota K. Liu J. Maldonado M. Hyle Park B. Nam J. (2018). Magnitude‐dependent and Inversely‐related Osteogenic/chondrogenic Differentiation of Human Mesenchymal Stem Cells under Dynamic Compressive Strain. J. Tissue Eng. Regen. Med. 12 (2), e637–e647. 10.1002/term.2332 27688005

[B47] Horner C. B. Maldonado M. Tai Y. Rony R. M. I. K. Nam J. (2019). Spatially Regulated Multiphenotypic Differentiation of Stem Cells in 3D via Engineered Mechanical Gradient. ACS Appl. Mater. Inter. 11 (49), 45479–45488. 10.1021/acsami.9b17266 31714732

[B48] Hu K. Sun H. Gui B. Sui C. (2017). TRPV4 Functions in Flow Shear Stress Induced Early Osteogenic Differentiation of Human Bone Marrow Mesenchymal Stem Cells. Biomed. Pharmacother. 91, 841–848. 10.1016/j.biopha.2017.04.094 28501773

[B49] Huang C. Holfeld J. Schaden W. Orgill D. Ogawa R. (2013). Mechanotherapy: Revisiting Physical Therapy and Recruiting Mechanobiology for a new era in Medicine. Trends Mol. Med. 19 (9), 555–564. 10.1016/j.molmed.2013.05.005 23790684

[B50] Hui C. F. F. Chan C. W. Yeung H. Y. Lee K. M. Qin L. Li G. (2011). Low-intensity Pulsed Ultrasound Enhances Posterior Spinal Fusion Implanted with Mesenchymal Stem Cells-Calcium Phosphate Composite without Bone Grafting. Spine 36 (13), 1010–1016. 10.1097/BRS.0b013e318205c5f5 21325987

[B51] Hunziker E. B. (2002). Articular Cartilage Repair: Basic Science and Clinical Progress. A Review of the Current Status and Prospects. Osteoarthritis and Cartilage 10 (6), 432–463. 10.1053/joca.2002.0801 12056848

[B52] Jepsen D. B. Ryg J. Hansen S. Jørgensen N. R. Gram J. Masud T. (2019). The Combined Effect of Parathyroid Hormone (1-34) and Whole-Body Vibration Exercise in the Treatment of Postmenopausal OSteoporosis (PaVOS Study): a Randomized Controlled Trial. Osteoporos. Int. 30 (9), 1827–1836. 10.1007/s00198-019-05029-z 31309239 PMC6717187

[B53] Jessop H. L. Suswillo R. F. Rawlinson S. C. Zaman G. Lee K. Das-Gupta V. (2004). Osteoblast-Like Cells from Estrogen Receptor α Knockout Mice Have Deficient Responses to Mechanical Strain. J. Bone Miner Res. 19 (6), 938–946. 10.1359/jbmr.2004.19.6.938 15190886

[B54] Jiang Y. N. Zhao J. Chu F. T. Jiang Y. Y. Tang G. H. (2018). Tension-loaded Bone Marrow Stromal Cells Potentiate the Paracrine Osteogenic Signaling of Co-cultured Vascular Endothelial Cells. Biol. Open 7 (6), bio032482. 10.1242/bio.032482 29716948 PMC6031349

[B55] Johnson G. P. Fair S. Hoey D. A. (2021). Primary Cilium-Mediated MSC Mechanotransduction Is Dependent on Gpr161 Regulation of Hedgehog Signalling. Bone 145, 115846. 10.1016/j.bone.2021.115846 33450431

[B56] Johnson G. P. Stavenschi E. Eichholz K. F. Corrigan M. A. Fair S. Hoey D. A. (2018). Mesenchymal Stem Cell Mechanotransduction Is cAMP Dependent and Regulated by Adenylyl Cyclase 6 and the Primary Cilium. J. Cel Sci 131 (21), jcs222737. 10.1242/jcs.222737 30301777

[B57] Kechagia J. Z. Ivaska J. Roca-Cusachs P. (2019). Integrins as Biomechanical Sensors of the Microenvironment. Nat. Rev. Mol. Cel Biol 20 (8), 457–473. 10.1038/s41580-019-0134-2 31182865

[B58] Kirby T. J. Lammerding J. (2018). Emerging Views of the Nucleus as a Cellular Mechanosensor. Nat. Cel Biol 20 (4), 373–381. 10.1038/s41556-018-0038-y PMC6440800 29467443

[B59] Kuhn N. Z. Tuan R. S. (2010). Regulation of Stemness and Stem Cell Niche of Mesenchymal Stem Cells: Implications in Tumorigenesis and Metastasis. J. Cel. Physiol. 222 (2), 268–277. 10.1002/jcp.21940 19847802

[B60] Kusuyama J. Bandow K. Shamoto M. Kakimoto K. Ohnishi T. Matsuguchi T. (2014). Low Intensity Pulsed Ultrasound (LIPUS) Influences the Multilineage Differentiation of Mesenchymal Stem and Progenitor Cell Lines through ROCK-Cot/Tpl2-MEK-ERK Signaling Pathway. J. Biol. Chem. 289 (15), 10330–10344. 10.1074/jbc.M113.546382 24550383 PMC4036157

[B61] Kwon R. Y. Meays D. R. Tang W. J. Frangos J. A. (2010). Microfluidic Enhancement of Intramedullary Pressure Increases Interstitial Fluid Flow and Inhibits Bone Loss in Hindlimb Suspended Mice. J. Bone Miner Res. 25 (8), 1798–1807. 10.1002/jbmr.74 20200992 PMC3153350

[B62] Lanyon L. Baggott D. (1976). Mechanical Function as an Influence on the Structure and Form of Bone. The J. Bone Jt. Surg. Br. volume 58-B (4), 436–443. 10.1302/0301-620X.58B4.1018029 1018029

[B63] Lee H. J. Diaz M. F. Ewere A. Olson S. D. Cox C. S. Jr. Wenzel P. L. (2017). Focal Adhesion Kinase Signaling Regulates Anti-inflammatory Function of Bone Marrow Mesenchymal Stromal Cells Induced by Biomechanical Force. Cell Signal. 38, 1–9. 10.1016/j.cellsig.2017.06.012 28647573 PMC5548629

[B64] Lee W. Leddy H. A. Chen Y. Lee S. H. Zelenski N. A. McNulty A. L. (2014). Synergy between Piezo1 and Piezo2 Channels Confers High-Strain Mechanosensitivity to Articular Cartilage. Proc. Natl. Acad. Sci. USA 111 (47), E5114–E5122. 10.1073/pnas.1414298111 25385580 PMC4250098

[B65] Li C.-J. Madhu V. Balian G. Dighe A. S. Cui Q. (2015a). Cross-Talk between VEGF and BMP-6 Pathways Accelerates Osteogenic Differentiation of Human Adipose-Derived Stem Cells. J. Cel. Physiol. 230 (11), 2671–2682. 10.1002/jcp.24983 25753222

[B66] Li C. W. Lau Y. T. Lam K. L. Chan B. P. (2020). Mechanically Induced Formation and Maturation of 3D-Matrix Adhesions (3DMAs) in Human Mesenchymal Stem Cells. Biomaterials 258, 120292. 10.1016/j.biomaterials.2020.120292 32818825

[B67] Li D. Tang T. Lu J. Dai K. (2009). Effects of Flow Shear Stress and Mass Transport on the Construction of a Large-Scale Tissue-Engineered Bone in a Perfusion Bioreactor. Tissue Eng. Part A 15 (10), 2773–2783. 10.1089/ten.TEA.2008.0540 19226211

[B68] Li H. Wu W. He X. Cao C. Yu X. Zeng Y. (2019a). Applying Vibration in Early Postmenopausal Osteoporosis Promotes Osteogenic Differentiation of Bone Marrow-Derived Mesenchymal Stem Cells and Suppresses Postmenopausal Osteoporosis Progression. Biosci. Rep. 39 (9). 10.1042/bsr20191011 PMC6722487 31406012

[B69] Li J. Hou B. Tumova S. Muraki K. Bruns A. Ludlow M. J. (2014). Piezo1 Integration of Vascular Architecture with Physiological Force. Nature 515 (7526), 279–282. 10.1038/nature13701 25119035 PMC4230887

[B70] Li J. Hu C. Han L. Liu L. Jing W. Tang W. (2015b). MiR-154-5p Regulates Osteogenic Differentiation of Adipose-Derived Mesenchymal Stem Cells under Tensile Stress through the Wnt/PCP Pathway by Targeting Wnt11. Bone 78, 130–141. 10.1016/j.bone.2015.05.003 25959411

[B71] Li R. Liang L. Dou Y. Huang Z. Mo H. Wang Y. (2015c). Mechanical Stretch Inhibits Mesenchymal Stem Cell Adipogenic Differentiation through TGFβ1/Smad2 Signaling. J. Biomech. 48 (13), 3656–3662. 10.1016/j.jbiomech.2015.08.013 26341460

[B72] Li X. Han L. Nookaew I. Mannen E. Silva M. J. Almeida M. (2019b). Stimulation of Piezo1 by Mechanical Signals Promotes Bone Anabolism. Elife 8. 10.7554/eLife.49631 PMC6779475 31588901

[B74] Liu L. Liu M. Li R. Liu H. Du L. Chen H. (2017). MicroRNA-503-5p Inhibits Stretch-Induced Osteogenic Differentiation and Bone Formation. Cell Biol Int 41 (2), 112–123. 10.1002/cbin.10704 27862699

[B75] Liu L. Yu B. Chen J. Tang Z. Zong C. Shen D. (2012). Different Effects of Intermittent and Continuous Fluid Shear Stresses on Osteogenic Differentiation of Human Mesenchymal Stem Cells. Biomech. Model. Mechanobiol 11 (3-4), 391–401. 10.1007/s10237-011-0319-x 21633819

[B76] Loebel C. Mauck R. L. Burdick J. A. (2019). Local Nascent Protein Deposition and Remodelling Guide Mesenchymal Stromal Cell Mechanosensing and Fate in Three-Dimensional Hydrogels. Nat. Mater. 18 (8), 883–891. 10.1038/s41563-019-0307-6 30886401 PMC6650309

[B77] Lu Y. Zhao Q. Liu Y. Zhang L. Li D. Zhu Z. (2018). Vibration Loading Promotes Osteogenic Differentiation of Bone Marrow-Derived Mesenchymal Stem Cells via P38 MAPK Signaling Pathway. J. Biomech. 71, 67–75. 10.1016/j.jbiomech.2018.01.039 29503016

[B78] Lv P.-y. Gao P.-f. Tian G.-j. Yang Y.-y. Mo F.-f. Wang Z.-h. (2020). Osteocyte-derived Exosomes Induced by Mechanical Strain Promote Human Periodontal Ligament Stem Cell Proliferation and Osteogenic Differentiation via the miR-181b-5p/PTEN/AKT Signaling Pathway. Stem Cel Res Ther 11 (1), 295. 10.1186/s13287-020-01815-3 PMC7367226 32680565

[B79] Manokawinchoke J. Pavasant P. Sawangmake C. Limjeerajarus N. Limjeerajarus C. N. Egusa H. (2019). Intermittent Compressive Force Promotes Osteogenic Differentiation in Human Periodontal Ligament Cells by Regulating the Transforming Growth Factor-β Pathway. Cell Death Dis 10 (10), 761. 10.1038/s41419-019-1992-4 31591384 PMC6779887

[B80] McCoy R. J. Widaa A. Watters K. M. Wuerstle M. Stallings R. L. Duffy G. P. (2013). Orchestrating Osteogenic Differentiation of Mesenchymal Stem Cells-Identification of Placental Growth Factor as a Mechanosensitive Gene with a Pro-osteogenic Role. Stem Cells 31 (11), 2420–2431. 10.1002/stem.1482 23897668

[B81] McDermott A. M. Herberg S. Mason D. E. Collins J. M. Pearson H. B. Dawahare J. H. (2019). Recapitulating Bone Development through Engineered Mesenchymal Condensations and Mechanical Cues for Tissue Regeneration. Sci. Transl. Med. 11 (495), eaav7756. 10.1126/scitranslmed.aav7756 31167930 PMC6959418

[B82] Michalopoulos E. Knight R. L. Korossis S. Kearney J. N. Fisher J. Ingham E. (2012). Development of Methods for Studying the Differentiation of Human Mesenchymal Stem Cells under Cyclic Compressive Strain. Tissue Eng. C: Methods 18 (4), 252–262. 10.1089/ten.TEC.2011.0347 PMC3311877 22047076

[B83] Minematsu A. Nishii Y. Imagita H. Sakata S. (2019). Possible Effects of Whole Body Vibration on Bone Properties in Growing Rats. Osteoporos. Sarcopenia 5 (3), 78–83. 10.1016/j.afos.2019.07.001 31728424 PMC6838745

[B84] Moore K. A. Lemischka I. R. (2006). Stem Cells and Their Niches. Science 311 (5769), 1880–1885. 10.1126/science.1110542 16574858

[B85] Nardone G. Oliver-De La Cruz J. Vrbsky J. Martini C. Pribyl J. Skládal P. (2017). YAP Regulates Cell Mechanics by Controlling Focal Adhesion Assembly. Nat. Commun. 8, 15321. 10.1038/ncomms15321 28504269 PMC5440673

[B86] Nathan A. S. Baker B. M. Nerurkar N. L. Mauck R. L. (2011). Mechano-topographic Modulation of Stem Cell Nuclear Shape on Nanofibrous Scaffolds. Acta Biomater. 7 (1), 57–66. 10.1016/j.actbio.2010.08.007 20709198 PMC2967658

[B87] Pathak M. M. Nourse J. L. Tran T. Hwe J. Arulmoli J. Le D. T. T. (2014). Stretch-activated Ion Channel Piezo1 Directs Lineage Choice in Human Neural Stem Cells. Proc. Natl. Acad. Sci. USA 111 (45), 16148–16153. 10.1073/pnas.1409802111 25349416 PMC4234578

[B88] Pelaez D. Arita N. Cheung H. S. (2012). Extracellular Signal-Regulated Kinase (ERK) Dictates Osteogenic And/or Chondrogenic Lineage Commitment of Mesenchymal Stem Cells under Dynamic Compression. Biochem. Biophysical Res. Commun. 417 (4), 1286–1291. 10.1016/j.bbrc.2011.12.131 22240026

[B89] Pongkitwitoon S. Uzer G. Rubin J. Judex S. (2016). Cytoskeletal Configuration Modulates Mechanically Induced Changes in Mesenchymal Stem Cell Osteogenesis, Morphology, and Stiffness. Sci. Rep. 6, 34791. 10.1038/srep34791 27708389 PMC5052530

[B90] Price C. Zhou X. Li W. Wang L. (2011). Real-time Measurement of Solute Transport within the Lacunar-Canalicular System of Mechanically Loaded Bone: Direct Evidence for Load-Induced Fluid Flow. J. Bone Miner Res. 26 (2), 277–285. 10.1002/jbmr.211 20715178 PMC3179346

[B91] Pugin J. Dunn I. Jolliet P. Tassaux D. Magnenat J.-L. Nicod L. P. (1998). Activation of Human Macrophages by Mechanical Ventilation *In Vitro* . Am. J. Physiology-Lung Cell Mol. Physiol. 275 (6), L1040–L1050. 10.1152/ajplung.1998.275.6.L1040 9843840

[B92] Qi W. Yan Y.-B. Lei W. Wu Z.-X. Zhang Y. Liu D. (2012). Prevention of Disuse Osteoporosis in Rats by Cordyceps Sinensis Extract. Osteoporos. Int. 23 (9), 2347–2357. 10.1007/s00198-011-1842-4 22159671

[B93] Ravichandran A. Lim J. Chong M. S. K. Wen F. Liu Y. Pillay Y. T. (2017). *In Vitro* cyclic Compressive Loads Potentiate Early Osteogenic Events in Engineered Bone Tissue. J. Biomed. Mater. Res. 105 (8), 2366–2375. 10.1002/jbm.b.33772 27527120

[B94] Robling A. G. Bonewald L. F. (2020). The Osteocyte: New Insights. Annu. Rev. Physiol. 82, 485–506. 10.1146/annurev-physiol-021119-034332 32040934 PMC8274561

[B95] Robling A. G. Hinant F. M. Burr D. B. Turner C. H. (2002). Improved Bone Structure and Strength after Long-Term Mechanical Loading Is Greatest if Loading Is Separated into Short Bouts. J. Bone Miner Res. 17 (8), 1545–1554. 10.1359/jbmr.2002.17.8.1545 12162508

[B96] Ruehle M. A. Eastburn E. A. LaBelle S. A. Krishnan L. Weiss J. A. Boerckel J. D. (2020). Extracellular Matrix Compression Temporally Regulates Microvascular Angiogenesis. Sci. Adv. 6 (34), eabb6351. 10.1126/sciadv.abb6351 32937368 PMC7442478

[B97] Saxon L. K. Robling A. G. Alam I. Turner C. H. (2005). Mechanosensitivity of the Rat Skeleton Decreases after a Long Period of Loading, but Is Improved with Time off. Bone 36 (3), 454–464. 10.1016/j.bone.2004.12.001 15777679

[B98] Schandelmaier S. Kaushal A. Lytvyn L. Heels-Ansdell D. Siemieniuk R. A. Agoritsas T. (2017). Low Intensity Pulsed Ultrasound for Bone Healing: Systematic Review of Randomized Controlled Trials. Bmj 356, j656. 10.1136/bmj.j656 28348110 PMC5484179

[B99] Schoenenberger A. D. Tempfer H. Lehner C. Egloff J. Mauracher M. Bird A. (2020). Macromechanics and Polycaprolactone Fiber Organization Drive Macrophage Polarization and Regulate Inflammatory Activation of Tendon *In Vitro* and *In Vivo* . Biomaterials 249, 120034. 10.1016/j.biomaterials.2020.120034 32315865

[B100] Schreivogel S. Kuchibhotla V. Knaus P. Duda G. N. Petersen A. (2019). Load‐induced Osteogenic Differentiation of Mesenchymal Stromal Cells Is Caused by Mechano‐regulated Autocrine Signaling. J. Tissue Eng. Regen. Med. 13 (11), 1992–2008. 10.1002/term.2948 31359634

[B101] Shi Y. Li H. Zhang X. Fu Y. Huang Y. Lui P. P. Y. (2011). Continuous Cyclic Mechanical Tension Inhibited Runx2 Expression in Mesenchymal Stem Cells through RhoA-Erk1/2 Pathway. J. Cel. Physiol. 226 (8), 2159–2169. 10.1002/jcp.22551 21520068

[B102] Siller-Jackson A. J. Burra S. Gu S. Xia X. Bonewald L. F. Sprague E. (2008). Adaptation of Connexin 43-hemichannel Prostaglandin Release to Mechanical Loading. J. Biol. Chem. 283 (39), 26374–26382. 10.1074/jbc.M803136200 18676366 PMC2546557

[B103] Sittichokechaiwut A. Edwards J. H. Edwards J. Scutt A. Reilly G. (2010). Short Bouts of Mechanical Loading Are as Effective as Dexamethasone at Inducing Matrix Production by Human Bone Marrow Mesenchymal Stem Cells. eCM 20, 45–57. 10.22203/ecm.v020a05 20648425

[B104] Stavenschi E. Labour M.-N. Hoey D. A. (2017). Oscillatory Fluid Flow Induces the Osteogenic Lineage Commitment of Mesenchymal Stem Cells: The Effect of Shear Stress Magnitude, Frequency, and Duration. J. Biomech. 55, 99–106. 10.1016/j.jbiomech.2017.02.002 28256244

[B105] Steinmetz N. J. Aisenbrey E. A. Westbrook K. K. Qi H. J. Bryant S. J. (2015). Mechanical Loading Regulates Human MSC Differentiation in a Multi-Layer Hydrogel for Osteochondral Tissue Engineering. Acta Biomater. 21, 142–153. 10.1016/j.actbio.2015.04.015 25900444

[B106] Sugimoto A. Miyazaki A. Kawarabayashi K. Shono M. Akazawa Y. Hasegawa T. (2017). Piezo Type Mechanosensitive Ion Channel Component 1 Functions as a Regulator of the Cell Fate Determination of Mesenchymal Stem Cells. Sci. Rep. 7 (1), 17696. 10.1038/s41598-017-18089-0 29255201 PMC5735093

[B107] Sumanasinghe R. D. Pfeiler T. W. Monteiro-Riviere N. A. Loboa E. G. (2009). Expression of Proinflammatory Cytokines by Human Mesenchymal Stem Cells in Response to Cyclic Tensile Strain. J. Cel. Physiol. 219 (1), 77–83. 10.1002/jcp.21653 19089992

[B108] Swain S. M. Liddle R. A. (2021). Piezo1 Acts Upstream of TRPV4 to Induce Pathological Changes in Endothelial Cells Due to Shear Stress. J. Biol. Chem. 296, 100171. 10.1074/jbc.RA120.015059 33298523 PMC7948745

[B109] Tan J. Xu X. Tong Z. Lin J. Yu Q. Lin Y. (2015). Decreased Osteogenesis of Adult Mesenchymal Stem Cells by Reactive Oxygen Species under Cyclic Stretch: a Possible Mechanism of Age Related Osteoporosis. Bone Res. 3, 15003. 10.1038/boneres.2015.3 26273536 PMC4413016

[B110] Thompson M. Woods K. Newberg J. Oxford J. T. Uzer G. (2020). Low-intensity Vibration Restores Nuclear YAP Levels and Acute YAP Nuclear Shuttling in Mesenchymal Stem Cells Subjected to Simulated Microgravity. NPJ Microgravity 6 (1), 35. 10.1038/s41526-020-00125-5 33298964 PMC7708987

[B111] Timmins P. A. Wall J. C. (1977). Bone Water. Calc. Tis Res. 23 (1), 1–5. 10.1007/bf02012759 890540

[B112] Uda Y. Azab E. Sun N. Shi C. Pajevic P. D. (2017). Osteocyte Mechanobiology. Curr. Osteoporos. Rep. 15 (4), 318–325. 10.1007/s11914-017-0373-0 28612339 PMC5656287

[B113] Uddin S. M. Z. Qin Y.-X. (2013). Enhancement of Osteogenic Differentiation and Proliferation in Human Mesenchymal Stem Cells by a Modified Low Intensity Ultrasound Stimulation under Simulated Microgravity. PLoS One 8 (9), e73914. 10.1371/journal.pone.0073914 24069248 PMC3772078

[B114] Uzer G. Bas G. Sen B. Xie Z. Birks S. Olcum M. (2018). Sun-mediated Mechanical LINC between Nucleus and Cytoskeleton Regulates βcatenin Nuclear Access. J. Biomech. 74, 32–40. 10.1016/j.jbiomech.2018.04.013 29691054 PMC5962429

[B115] Vafaei R. Nassiri S. M. Siavashi V. (2017). β3‐Adrenergic Regulation of EPC Features through Manipulation of the Bone Marrow MSC Niche. J. Cel. Biochem. 118 (12), 4753–4761. 10.1002/jcb.26143 28513874

[B116] Wan M. Li C. Zhen G. Jiao K. He W. Jia X. (2012). Injury‐Activated Transforming Growth Factor β Controls Mobilization of Mesenchymal Stem Cells for Tissue Remodeling. Stem Cells 30 (11), 2498–2511. 10.1002/stem.1208 22911900 PMC3479365

[B117] Wang C. Shan S. Wang C. Wang J. Li J. Hu G. (2017). Mechanical Stimulation Promote the Osteogenic Differentiation of Bone Marrow Stromal Cells through Epigenetic Regulation of Sonic Hedgehog. Exp. Cel Res. 352 (2), 346–356. 10.1016/j.yexcr.2017.02.021 28215635

[B118] Wang L. You X. Lotinun S. Zhang L. Wu N. Zou W. (2020). Mechanical Sensing Protein PIEZO1 Regulates Bone Homeostasis via Osteoblast-Osteoclast Crosstalk. Nat. Commun. 11 (1), 282. 10.1038/s41467-019-14146-6 31941964 PMC6962448

[B119] Wang X. Li X. Li J. Zhai L. Liu D. Abdurahman A. (2021). Mechanical Loading Stimulates Bone Angiogenesis through Enhancing Type H Vessel Formation and Downregulating Exosomal miR‐214‐3p from Bone Marrow‐derived Mesenchymal Stem Cells. FASEB j. 35 (1), e21150. 10.1096/fj.202001080RR 33161580 PMC7748991

[B120] Wei F.-Y. Chow S. K. Chow S. Leung K.-S. Qin J. Guo A. (2016). Low-magnitude High-Frequency Vibration Enhanced Mesenchymal Stem Cell Recruitment in Osteoporotic Fracture Healing through the SDF-1/CXCR4 Pathway. eCM 31, 341–354. 10.22203/ecm.v031a22 27215741

[B121] Wei F.-Y. Leung K.-S. Li G. Qin J. Chow S. K.-H. Huang S. (2014). Low Intensity Pulsed Ultrasound Enhanced Mesenchymal Stem Cell Recruitment through Stromal Derived Factor-1 Signaling in Fracture Healing. PLoS One 9 (9), e106722. 10.1371/journal.pone.0106722 25181476 PMC4152330

[B122] Woo S. L. Kuei S. C. Amiel D. Gomez M. A. Hayes W. C. White F. C. (1981). The Effect of Prolonged Physical Training on the Properties of Long Bone. J. Bone Jt. Surg. 63 (5), 780–787. 10.2106/00004623-198163050-00013 7240300

[B123] Wysocki A. Butler M. Shamliyan T. Kane R. L. (2011). Whole-body Vibration Therapy for Osteoporosis: State of the Science. Ann. Intern. Med. 155 (10), 680w206–686613. 10.7326/0003-4819-155-10-201111150-00006 22084334

[B124] Xiang Y. H. Shao M. F. Song Y. Yang Z. Chen X. D. Fu Q. (2012). Effect of Cytoskeleton Reorganization Inhibition on the Activation of Extracellular Signal-Regulated Kinase in Osteoblasts by Fluid Shear Stress. Zhonghua Kou Qiang Yi Xue Za Zhi 47 (11), 680–683. 10.3760/cma.j.issn.1002-0098.2012.11.010 23302431

[B125] Xing Z. Lu C. Hu D. Yu Y.-y. Wang X. Colnot C. (2010). Multiple Roles for CCR2 during Fracture Healing. Dis. Model. Mech. 3 (7-8), 451–458. 10.1242/dmm.003186 20354109 PMC2898536

[B126] Xue R. Cartmell S. (2020). A Simple *In Vitro* Biomimetic Perfusion System for Mechanotransduction Study. Sci. Tech. Adv. Mater. 21 (1), 635–640. 10.1080/14686996.2020.1808432 PMC7534211 33061836

[B127] Yan Y. Wang L. Ge L. Pathak J. L. (2020). Osteocyte-Mediated Translation of Mechanical Stimuli to Cellular Signaling and its Role in Bone and Non-bone-related Clinical Complications. Curr. Osteoporos. Rep. 18 (1), 67–80. 10.1007/s11914-020-00564-9 31953640

[B128] Yoneda M. Suzuki H. Hatano N. Nakano S. Muraki Y. Miyazawa K. (2019). PIEZO1 and TRPV4, Which Are Distinct Mechano-Sensors in the Osteoblastic MC3T3-E1 Cells, Modify Cell-Proliferation. Ijms 20 (19), 4960. 10.3390/ijms20194960 PMC6801562 31597314

[B129] Yourek G. McCormick S. M. Mao J. J. Reilly G. C. (2010). Shear Stress Induces Osteogenic Differentiation of Human Mesenchymal Stem Cells. Regenerative Med. 5 (5), 713–724. 10.2217/rme.10.60 PMC4093787 20868327

[B130] Yu W. Chen C. Kou X. Sui B. Yu T. Liu D. (2021). Mechanical Force-Driven TNFα Endocytosis Governs Stem Cell Homeostasis. Bone Res. 8 (1), 44. 10.1038/s41413-020-00117-x 33384406 PMC7775432

[B131] Yue D. Zhang M. Lu J. Zhou J. Bai Y. Pan J. (2019). The Rate of Fluid Shear Stress Is a Potent Regulator for the Differentiation of Mesenchymal Stem Cells. J. Cel Physiol 234, 16312–16319. 10.1002/jcp.28296 30784070

[B132] Zhang H. Kay A. Forsyth N. R. Liu K.-K. El Haj A. J. (2012). Gene Expression of Single Human Mesenchymal Stem Cell in Response to Fluid Shear. J. Tissue Eng. 3 (1), 204173141245198. 10.1177/2041731412451988 PMC3394398 22798982

[B133] Zhang P. Liu X. Guo P. Li X. He Z. Li Z. (2021). Effect of Cyclic Mechanical Loading on Immunoinflammatory Microenvironment in Biofabricating Hydroxyapatite Scaffold for Bone Regeneration. Bioactive Mater. 6 (10), 3097–3108. 10.1016/j.bioactmat.2021.02.024 PMC7960680 33778191

[B134] Zhang Y. P. Qin F. Wu C. J. Li Y. R. Chen R. Shao M. F. (2009). Effects of LIMK2 RNA Interference on the Mechanosensitivity of C-Fos Gene in Osteoblast. Zhonghua Yi Xue Za Zhi 89 (44), 3143–3146. 10.3760/cma.j.issn.0376-2491.2009.44.012 20193279

[B135] Zhao W. Tang Y. Yang Y. Wang M. Yu H. (2019). Low-Magnitude, High-Frequency Vibration Promotes Osteogenic Differentiation via Intensifying miRNA-335-5p Expression. J. Environ. Pathol. Toxicol. Oncol. 38 (3), 271–283. 10.1615/JEnvironPatholToxicolOncol.2019030625 31679313

[B136] Zhou Y. Guan X. Guan X. Zhu Z. Gao S. Zhang C. (2011). Osteogenic Differentiation of Bone Marrow-Derived Mesenchymal Stromal Cells on Bone-Derived Scaffolds: Effect of Microvibration and Role of ERK1/2 Activation. eCM 22, 12–25. 10.22203/ecm.v022a02 21732279

